# Neural Circuits Underlying Social Fear in Rodents: An Integrative Computational Model

**DOI:** 10.3389/fnsys.2022.841085

**Published:** 2022-03-08

**Authors:** Valerio Alfieri, Andrea Mattera, Gianluca Baldassarre

**Affiliations:** Institute of Cognitive Sciences and Technologies, National Research Council, Rome, Italy

**Keywords:** social fear, social anxiety disorder, ventromedial hypothalamus, medial prefrontal cortex, computational neuroscience

## Abstract

Social avoidance in rodents arises from a complex interplay between the prefrontal cortex and subcortical structures, such as the ventromedial hypothalamus and the dorsal periaqueductal gray matter. Experimental studies are revealing the contribution of these areas, but an integrative view and model of how they interact to produce adaptive behavior are still lacking. Here, we present a computational model of social avoidance, proposing a set of integrated hypotheses on the possible macro organization of the brain system underlying this phenomenon. The model is validated by accounting for several different empirical findings and produces predictions to be tested in future experiments.

## 1. Introduction

The social anxiety disorder (SAD) is the third most frequent psychiatric disorder, also representing a risk factor for depression and addiction (Cohen et al., 2007; Stein and Stein, 2008; Leichsenring and Leweke, 2017; Dos Santos et al., 2019). In humans, SAD has been linked to alterations in the activity of prefrontal cortex, hyper-activation of the amygdala, and disfunctions in many other subcortical regions, such as the bed nucleus of the stria terminalis (BNST), the striatum and the periacqueductal gray matter (PAG) (Berkowitz et al., 2007; Goldin et al., 2009; Labuschagne et al., 2012; Arnold Anteraper et al., 2014; Duval et al., 2015; Marazziti et al., 2015; Stein, 2015; Clauss et al., 2019).

Even though cognitive behavioral therapy is an effective solution for the treatment of SAD, it has limitations concerning the individual costs and the timing of the therapy (Pilling et al., 2013; Mayo-Wilson et al., 2014; Scaini et al., 2016; Dos Santos et al., 2019). Evidence suggests that pharmacological medications could complement a psychological approach, especially in cases when the severity of the impairment could cause other psychological and health risks, such as depression and suicide attempts (Hambrick et al., 2003; Vitiello, 2009; Kelly et al., 2014; Rao and Andrade, 2017). Unfortunately, current pharmacological treatments are based on serotonin and norepinephrine reuptake inhibitors, presenting multiple collateral effects and requiring at least some weeks to obtain a therapeutic response (Dos Santos et al., 2019). Animal research is very helpful for understanding the pathogenesis of SAD and for the development of effective drugs for its treatment (Wang et al., 2020). Indeed, animals characterised by rich inter-specific social interactions usually exhibit a high level of behavioral flexibility. Social behavior often entails repeated encounters between antagonist individuals. These interactions cause the defeated animals to experience different levels of physical and psychosocial stress and promote the adjustment of behavior to suitably cope with future encounters (Chen and Hong, 2018; Diaz and Lin, 2020). The shift toward a social avoidance strategy is presumably an adaptive mechanism aimed to diminish future harm and facilitate alternative routes to the attainment of essential resources (Christoffel et al., 2015).

The translational value of murine research has long been undermined by the lack of paradigms capable of reproducing the core symptoms of SAD without confounding effects. Indeed, most protocols also induce unspecific symptoms, e.g., anhedonia, general anxiety, and impaired locomotory activity (Huhman, 2006; Wang et al., 2020). In the last decade, however, it has been discovered that two protocols overcome these limitations. In the classical social defeat protocol an aggressive conspecific is placed in the same cage of the experimental mouse to allow a non-lethal conflict. If this procedure is repeated no more than 3 days and for a few minutes only at a time (sub-chronic defeat protocol), it causes a specific social impairment without manifestation of anxiety, stress or altered exploratory behavior (Franklin et al., 2017). An alternative protocol to avoid unspecific symptoms, also known as social fear conditioning, consists of pairing the social investigation of a conspecific with a foot shock, akin to what is done in auditory fear conditioning (Toth et al., 2012, 2013). After these protocols the defeated animal displays avoidance of conspecifics, measured as more time spent freezing and in defensive postures, less time spent investigating the conspecific and a higher number of sudden retreats from the investigation (Toth et al., 2012, 2013; Franklin et al., 2017; Xu et al., 2019; Krzywkowski et al., 2020).

A growing amount of research has begun to dissect the neural substrates of social avoidance in rodents. This led to the identification of distinct brain regions involved in the processing of defensive responses to aggressive conspecifics and their dissociation from the parallel and mostly non-overlapping neural circuits encoding predatory fear or auditory fear conditioning (Gross and Canteras, 2012; Silva et al., 2013, 2016a). The ventrolateral part of the ventromedial hypothalamus (VMHvl) is considered to be a central hub for the coordination of behavioral responses directed toward conspecifics, such as aggression, mating, approach, and defense (Lin et al., 2011; Gross and Canteras, 2012; Sakurai et al., 2016). The VMHvl receives projections from the medial amygdala (MeA), that relays conspecific-related information from pheromonal and olfactory cues captured by the olfactory systems (Gross and Canteras, 2012; Silva et al., 2016a), and information on the spatial context from the ventral hippocampus (vHIP, Chang and Gean, 2019).

Calcium imaging studies revealed that sub-chronic social defeat induces phasic responses in a VMHvl neural population associated with an internal state of social threat (Krzywkowski et al., 2020). Subsequent exposure to the context where the defeat took place reactivates the same population that was recruited during the traumatic experience, suggesting that VMHvl encodes a fear engram (Krzywkowski et al., 2020). Moreover, optogenetic activation of the neurons that were active during the defeat is sufficient to enact fear manifestations (Sakurai et al., 2016). The downstream target of the VMHvl is the dorsal part of the periaqueductal gray (dPAG; Silva et al. 2013). This is a midbrain nucleus that orchestrates the defensive behavior by coordinating the structures responsible for performing the motor action.

Besides VMHvl and dPAG, another region involved in the regulation of social interactions is the medial prefrontal cortex (mPFC; Franklin et al. 2017; Xu et al. 2019; Wang et al. 2020). Modern studies aimed at explaining how cortical and subcortical systems interact to support social fear processes in rodents are drawing a puzzling picture. In particular, Franklin et al. (2017) found that sub-chronic social defeat induces a depotentiation of the connections from the mediodorsal thalamus (MDT) to the mPFC. In turn, the mPFC becomes less effective in the recruitment of the dPAG, resulting in social withdrawal. In agreement with this, the effect of sub-chronic social defeat can be mimicked through the inhibition of the output from the mPFC to the dPAG. This is in line with the experiments showing that subordinate mice in social ranking tests display reduced excitability in layer 5 neurons of the mPFC (Wang et al., 2011), whereas in dominant mice the connection between the MDT and the mPFC is potentiated (Zhou et al., 2017). Seemingly at odds with such evidence, Xu et al. (2019) found an increased firing of the pyramidal neurons in the mPFC during social avoidance. Consequently, lowering their activity through the manipulation of the upstream GABAergic neurons recovers social interactions (Xu et al., 2019). Overall, the fact that both an increase and a decrease of the activity of the mPFC excitatory neurons mediate social avoidance suggests that different sub-populations of the mPFC pyramidal neurons could exert an opposite influence on social behavior by acting as fear-ON and fear-OFF populations. Two lines of evidence further support this hypothesis. First, a study of aggressive behavior highlights a complex dual role of the mPFC, that is shown to be capable of inhibiting but also promoting aggression, possibly through the engagement of different sub-regions (Biro et al., 2017). Second, another study shows that during social exploration a neural population of the mPFC increases its activity while at the same time a second population turns off (Liang et al., 2018).

Reconciling these multiple pieces of evidence requires the integration of all the available information in the same framework to clarify the possible roles and interactions that cortical and subcortical structures express in social avoidance. The aim of this work is hence to present a system-level computational model able to aggregate the different experimental findings in a coherent scheme.

To our knowledge, while classical fear conditioning has been widely investigated with the use of computational models (Burgos and Murillo-Rodŕıguez, 2007; Mannella et al., 2008; Krasne et al., 2011; Anastasio, 2013; John et al., 2013; Moustafa et al., 2013; Carrere and Alexandre, 2015; Li et al., 2016; Bennett et al., 2019; Mattera et al., 2020), the model proposed here is the first to account for social avoidance. The model is based on a number of hypotheses, grounded on literature, concerning the involved brain areas, neural populations, and connections between them underlying social avoidance. The goal of the model construction is to operationalize such hypotheses and integrate them in a coherent whole (also giving a possible explanation to the “puzzling picture” illustrated above), to validate this with the qualitative reproduction of several current experiments, and then to derive new predictions testable in future experiments (Shen and McNaughton, 1996).

The rest of this article is organized as follows. First, we describe how we developed the model based on reasoned hypotheses grounded on the empirical literature. Second, we validate the model by reproducing some important experimental findings on sub-chronic social defeat and social fear conditioning. Third, we manipulate the model to produce new testable predictions. Last, we discuss our findings in light of the existing literature.

## 2. Methods

### 2.1. Firing Rate Units

**Figure 1:**
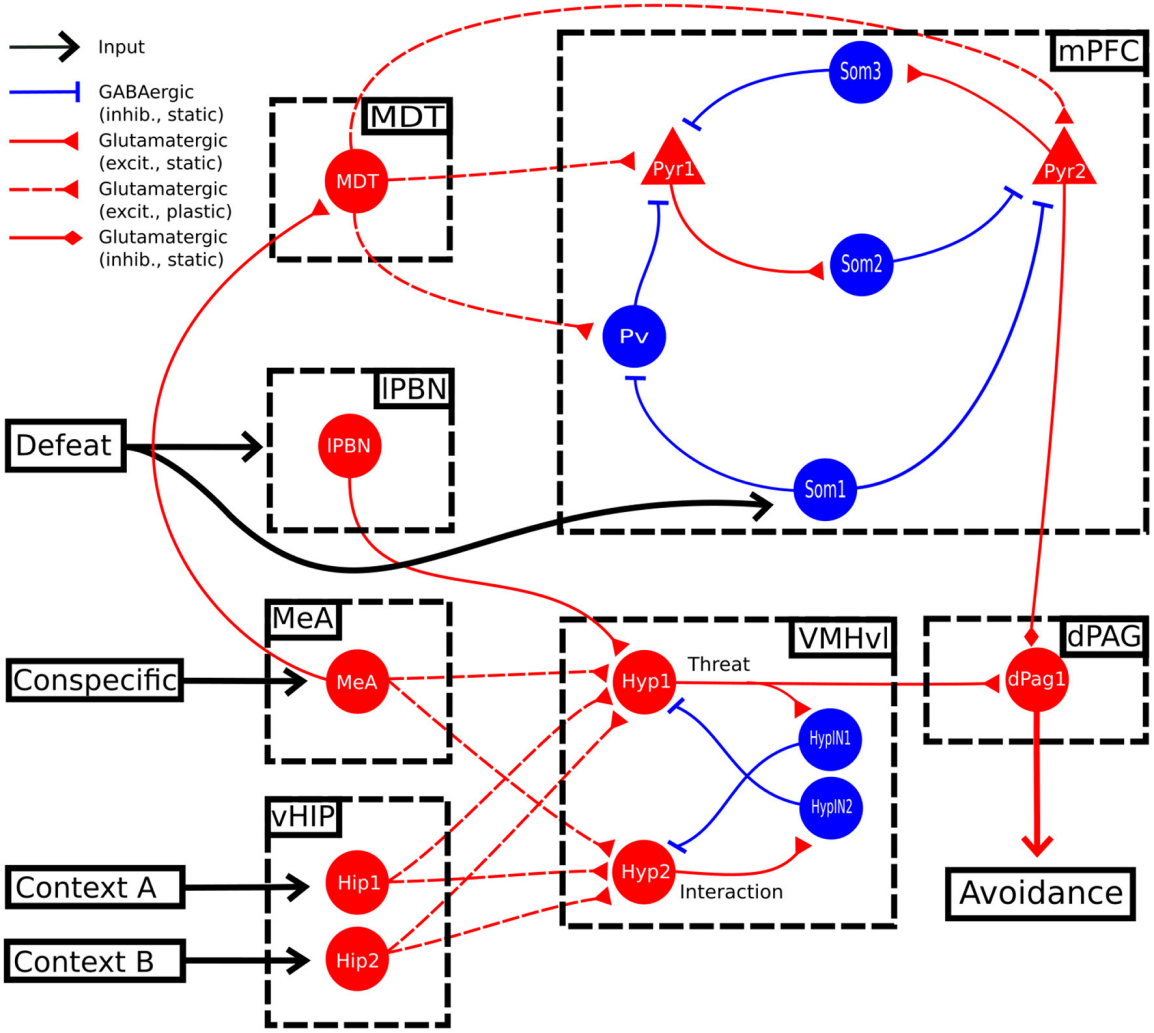
Model architecture. Scheme of the brain areas (dashed boxes), neural glutamatergic and GABAergic populations (red and blue lines, respectively), fixed and plastic connections (continuous and dashed lines, respectively), and inputs (black lines), forming the model. dPAG: dorsal periaqueductal gray matter; lPBN: lateral parabrachial nucleus; MeA: medial amygdala; MDT: mediodorsal thalamus; mPFC: medial prefrontal cortex; vHIP: ventral hippocampus; VMHvl: ventrolateral division of the ventromedial hypothalamus.

The model is formed by firing rate leaky units, each representing a population of neurons. The voltage *V*_*post*_ of a post-synaptic unit is regulated through the following differential equation:


(1) $$\begin{array}{r} \tau {\overset{∙}{V}}_{post}=-V_{post}+I+\underset{pre}{\sum }w_{post,pre}F\left(V_{pre}\right) \end{array}$$

where τ is the time constant, *I* is the external input to the unit (representing the “defeat,” the “conspecific,” and the “context;” see Figure 1), *w*_*post,pre*_ is the connection weight between the presynaptic unit *pre* and unit *post*. *F* is the activation of the unit, computed with the hyperbolic tangent function *tanh*(*x*), and represents the firing rate of a population of neurons (Burgos and Murillo-Rodŕıguez, 2007; Moustafa et al., 2013; Carrere and Alexandre, 2015; Mannella et al., 2016; Bennett et al., 2019; Mattera et al., 2020):


(2) $$\begin{array}{r} F\left(V\right)={\left[tanh\left(V\right)\right]}^{+} \end{array}$$

where [*x*]^+^ is the positive function ([*x*]^+^ = *x* if *x*≥0, and [*x*]^+^ = 0 if *x* < 0). The tanh function restricts the range of the units activation in the interval [0,1]. The equation was approximated with the Euler method with discrete time steps Δt (the values of the model parameters are listed in Supplementary Table S1).

### 2.2. Model Architecture

Figure 1 illustrates the brain areas and their connections forming the architecture of the model; these areas and connections are grounded in the biological literature on social fear. This architecture is not meant to be comprehensive of all the mouse brain areas involved in social behavior, but only of those necessary to explain the key target experiments of section 3.3. In the decision of which areas to include or not into the model we used the following “criteria of coherence” by selecting only studies that: (1) focused on mice; (2) investigated areas activated during an aversive social encounter (Silva et al., 2013; Krzywkowski et al., 2020) or after paradigms of social sub-chronic defeat or foot-shock social fear conditioning (Franklin et al. 2017; Xu et al. 2019; Krzywkowski et al. 2020; conditions that avoid non-specific symptoms and confounding factors) when the defeated mouse interacted with the conspecific. This allowed us to identify and simulate the murine brain areas recruited in naive mice at the time of sub-chronic social defeat, and those activated in defeated mice when they subsequently avoid the conspecifics. The information coming from those articles points out the VMHvl, dPAG and mPFC as areas strongly involved in the specific symptoms, thus we focused on them in our model. Knowledge gaps in the literature were filled by formulating specific hypotheses, that were subsequently validated with the reproduction of the target experiments.

The model receives three inputs, representing the conspecific, the spatial context, and the social defeat. The first input activates the MeA (Li et al., 2017; Nordman et al., 2020) and is related to the detection of sensory cues signaling the presence of a conspecific; the second input activates the vHIP that encodes spatial information about two possible alternative contexts through two neural populations, Hip1 and Hip2. Both the conspecific and contextual input reach two populations of the VMHvl (see below; Sakurai et al. 2016; Chang and Gean 2019; Wang et al. 2019; Falkner et al. 2020; Krzywkowski et al. 2020). The third input encodes the social defeat and activates the lateral parabrachial nucleus (lPBN), which in turn conveys excitatory pain-related signals to the VMHvl (Chiang et al., 2020). The VMHvl sends excitatory connections to the dPAG excitatory neural unit dPag1, which triggers a social avoidance behavioral output (Silva et al., 2013; Deng et al., 2016).

In the model, there are two units in the VMHvl, Hyp1 and Hyp2, whose activity is antagonistic. We modeled them on the basis of the results from Krzywkowski et al. (2020) who showed that, during the aversive social encounter, a population of VMHvl neurons increase its activity while another one decreases it (the authors called them “Defeat+” and “Defeat-.”) In the construction of the VMHvl connectivity, we hypothesized that the two excitatory neural populations within the VMHvl exhibit mutual lateral inhibition through local GABAergic interneurons (HypIN1 and HypIN2), effectively implementing a *winner-takes-all* mechanism corresponding to a competition to control behavior between the two populations. The presence of inhibitory interneurons within VMHvl has been proposed in previous studies (Choi et al., 2005; Kim et al., 2019; Lo et al., 2019).

In addition to VMHvl, the projections from the MeA reach the MDT (Krettek and Price, 1977; Canteras et al., 1995; Mitchell and Chakraborty, 2013). This relays multi-modal cognitive/emotional information to the cortex through plastic connections (Kuroda et al., 1998; Delevich et al., 2015; Franklin et al., 2017; Collins et al., 2018; Nelson et al., 2019).

In the construction of the mPFC, we followed the standard approach of the top-down models (John et al., 2013; Moustafa et al., 2013; Li et al., 2016; Oliva et al., 2018; Bennett et al., 2019; Mattera et al., 2020), abstracting over the layered circuits and intra-cortical connectivity of the cortex. In particular, since ours is a system-level model considering several areas, it was not possible to include in the model all the known connections linking those areas. We thus followed a parsimony principle and included in the model only the connections that were functionally relevant to actually capture the target experiments. Since, however, the necessity of such connections was still an open problem, we also performed a sensitivity analysis (see below) to highlight the importance of the considered connections. Analogously to what stated in Mannella et al. (2016), this approach has the following advantages: (a) the production of hypotheses about the identity and connectivity of the neural populations of the mPFC relevant for the targeted phenomenon, here social avoidance; (b) the identification of the knowledge gaps regarding the mechanisms of recruitment of those populations; and (c) the development of predictions based on them that can be empirically tested.

The authors of Xu et al. (2019) reported that sub-chronic social defeat leads to an increase of the firing of the mPFC pyramidal neurons, and that a stimulation of parvalbumin positive neurons (parvalbumin+), performed through the inhibition of the upstream somatostatin positive interneurons (somatostatin+), reduces their activity and reverts conditioning. We thus implemented in the model an excitatory neural population, Pyr1, targeted by a double-inhibition circuit formed by Pv and Som1, representing, respectively, parvalbumin+ and somatostatin+ neurons (Figure 1). Interestingly, auditory fear conditioning relies on a mPFC disinhibitory circuit analogous to the one described for social fear conditioning (Courtin et al., 2014; Cummings and Clem, 2020). In particular, it has been shown that the acquisition of auditory fear conditioning requires the transient inhibition of the parvalbumin+ interneurons (Courtin et al., 2014). It has been suggested that a similar recruitment of a disinhibitory microcircuit, through the activation of somatostatin+ interneurons upstream of the parvalbumin+ interneurons, could establish social fear in the mPFC (Wang et al., 2020). On this basis, in the model we hypothesised that sub-chronic social defeat recruits both the nociceptive center lPBN and the unit Som1 in the mPFC causing the disinhibition of Pyr1.

A model where social avoidance is driven, at the mPFC level, only by the activation of a single population of pyramidal neurons cannot explain the findings of Franklin et al. (2017), which found that a decrease in the layer 5 of the mPFC output to the dPAG leads to social avoidance. We thus hypothesized that another population of pyramidal neurons, besides Pyr1, is involved in sub-chronic social defeat. In the model, this population Pyr2 should inhibit social avoidance in an opposite way with respect to the Pyr1 population described by Xu et al. (2019). Supporting this claim, and in accordance to Franklin et al. (2017), a recent experimental study showed that the administration of the psychedelic lysergic acid diethylamide (LSD), which enhances excitatory burst firing, promotes social behavior in mice. Interestingly, the pro-social effect of LSD is prevented by the optogenetic inhibition of the mPFC pyramidal neurons (De Gregorio et al., 2021). The two units Pyr1 and Pyr2 are reciprocally connected by Som2 and Som3, representing two populations of putative somatostatin+ interneurons driving lateral inhibition (Kapfer et al., 2007; Silberberg and Markram, 2007; Riedemann, 2019).

The second hypothesis concerns the mPFC-dPAG projections. As Franklin et al. (2017) previously observed, prefrontal projections from pyramidal neurons located in layer 5 exert inhibitory control over the activity of the dPAG neurons. However, it has also been established that pyramidal cells projecting to the dPAG are glutamatergic and therefore the mechanism through which they exert the inhibitory control remains to be explained. In *ex vivo* circuit-mapping experiments, optogenetic-induced stimulation of pyramidal prefrontal projections to the dPAG induces short-latency excitatory currents in some Vglut2+ cells (13%), but not in the Vgat+ cell (Franklin et al., 2017). This suggests the absence of a feedforward GABAergic inhibition and the possibility of an indirect, long-latency suppression of incoming hypothalamic inputs driven by metabotropic glutamatergic receptors. For this reason, we simulated the prefrontal top-down control of Pyr2 on the dPag1 with a direct inhibition, thus abstracting over the specific mechanism supporting the process.

In addition to this “basic model,” we explored the possibility of an alternative model (**Figure 3**) where we connected another area, indicated as lateral septum (LS), to the threat unit Hyp1 of the VMHvl. The possibility of this interaction is supported by (a) anatomical tracing studies (Risold and Swanson, 1997) and (b) functional evidence showing that the optogenetic stimulation of the LS projections reaching VMHvl induces social investigation (Wong et al., 2016).

The weights of the connections of the two models (listed in Supplementary Table S2, together with the literature supporting their existence) were manually adjusted until we found a configuration sufficient to reproduce all the target experiments (Mattera et al., 2020). To test the robustness of the results to the modification of the parameters, we performed a sensitivity analysis (see Supplementary Material and Section 3.5). Given the absence of noise and of weight randomization the model is completely deterministic, so it was not needed to run the simulation multiple times with different seeds of the random number generator.

### 2.3. Synaptic Plasticity

Some of the connections of the models are fixed while others are plastic. The weights of the plastic connections are updated according to a simplified Bienenstock–Cooper–Munro (BCM) learning rule (Bienenstock et al., 1982):


(3) $$\begin{array}{r} \Delta W=\alpha \cdot \left(F_{post}-\theta \right)\cdot F_{pre} \end{array}$$

where α is the learning rate, θ is a threshold (Supplementary Table S1), and F_post_ and F_pre_ are the firing rates of the post- and pre-synaptic units. The plastic weights were clipped within a (*Wmax, Wmin*) range (Supplementary Table S1).

To decide which connections had to be plastic, we followed this strategy. First, we considered to be plastic all the connections that were found to be plastic in the experimental literature on the social behavior of mice (Supplementary Table S2). The rest were considered fixed. Finally, we ran the simulations to fit the experimental data. We observed that, to reproduce the experiment of Krzywkowski et al. (2020), the connection between vHIP and VMHvl had to be made plastic. This corresponds to a principle of parsimony aimed at simplifying the model, staying as much as possible grounded on literature, and minimizing the hypothesis.

### 2.4. Simulation Protocol

**Figure 2:**
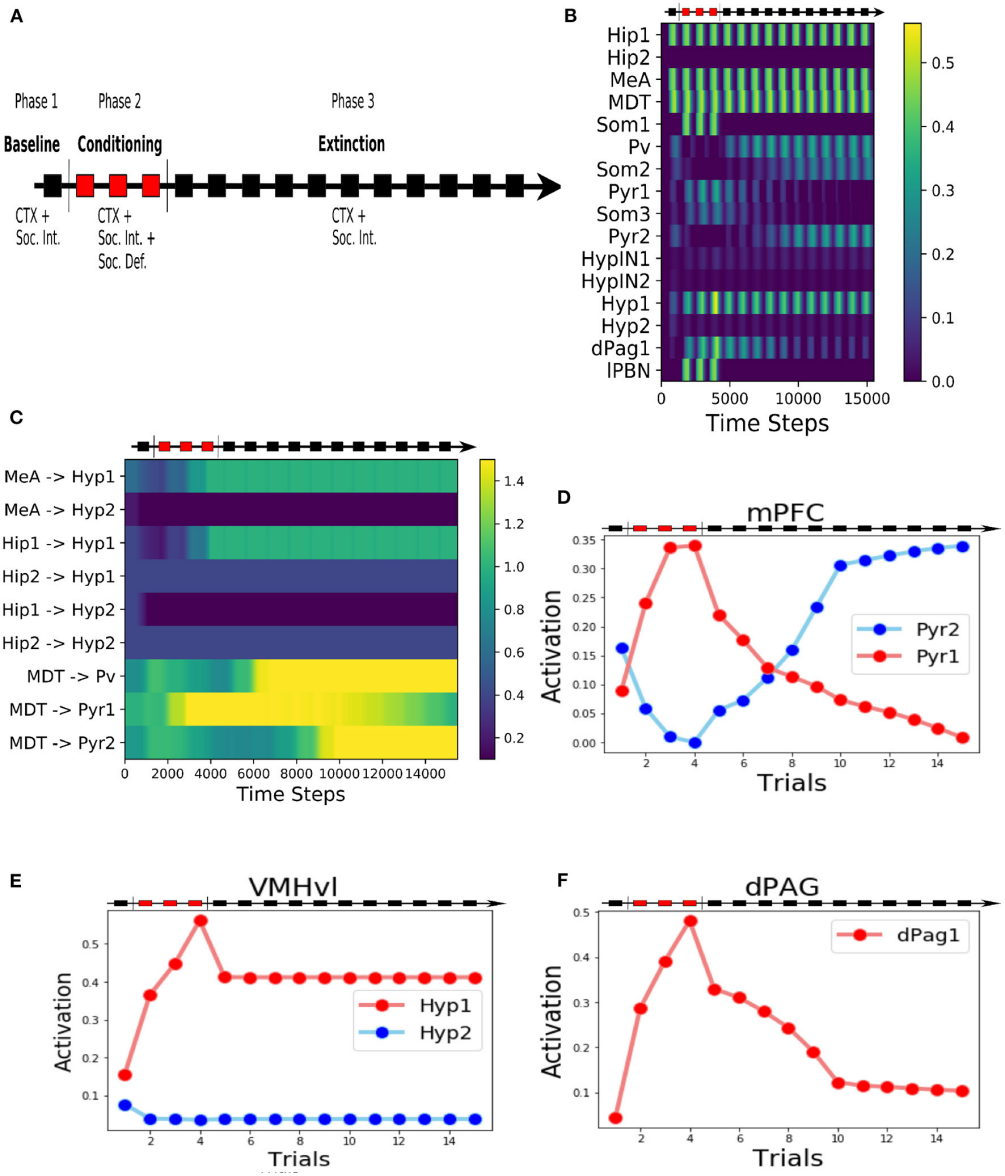
Behaviour of the model during the three-phases protocol. **(A)** The protocol of social fear conditioning and extinction, drawn from Franklin et al. (2017), Toth et al. (2012), and Ayash et al. (2020). **(B)** Heatmap of the activation of the model units during the protocol. **(C)** Heatmap showing the evolution of the model connection weights during the protocol. **(D)** Activation of the two excitatory units of the mPFC. **(E)** Activation of the two excitatory units in the VMHvl. **(F)** Activation of the dPag1, representing the output of the model.

In the model, the activation of the unit dPag1 represents social avoidance (Silva et al., 2013). To measure the baseline level of avoidance (Figure 2A), the model underwent a single trial (duration of 1 trial = 500 timesteps) of social interaction involving the presence of input to MeA (conspecific) and Hip1 (context). This was followed by three trials [as in Franklin et al. (2017)] of social conditioning (inputs to MeA and Hip1; defeat input to lPBN and Som1). Finally, these conditioning trials were followed by 11 trials [as in Toth et al. (2012)] of extinction (inputs to MeA and Hip1). This protocol is based on the foot-shock social conditioning and the sub-chronic defeat and aims to capture the common mechanisms underlying them. We cannot exclude that these 2 paradigms could induce plasticity at different sites. However, these 2 paradigms induce the same symptoms, measured in the three chamber apparatus (containing in one chamber the unfamiliar mouse to interact with) or in an open field with a cage containing the unfamiliar mouse (Toth et al., 2012; Franklin et al., 2017; Xu et al., 2019; Krzywkowski et al., 2020) and are considered the best available to recapitulate the symptoms of SAD (Wang et al., 2020). Other protocols, such as the chronic defeat (Huhman, 2006) or the juvenile isolation (Yamamuro et al., 2020), were not taken into account because of the non-specific symptoms (that could indicate different fear pathways) and the difficulty to figure which input units to activate into the model in order to reproduce them. For the rest of this article, we will use the terms “sub-chronic social defeat” and “social fear conditioning” in an interchangeable way to refer to our social fear protocol.

## 3. Results

### 3.1. The Overall Behavior of the Model

During the baseline (phase 1 of the protocol, Figure 2A), when the model was exposed only to the context (represented by the activation of the unit Hip1) and the conspecific (represented by the activation of the MeA), the hypothalamic unit Hyp1 (the threat unit, see Figures 1, 2B,E) and the mPFC excitatory units Pyr1 and Pyr2 were mildly activated (Figures 2B,D). The dPag1 unit remained inactive (Figure 2F).

During the three trials of conditioning (Phase 2 of the protocol, Figure 2A) we activated the units Som1 in mPFC and lPBN, concomitantly with Hip1 and MeA. This induced a reorganization of the weights at the cortical and subcortical levels (Figure 2C). In particular, at the subcortical level, the connection between MeA and the threat unit Hyp1 underwent LTP, while the connection between MeA and the interaction unit Hyp2 was depotentiated. At the cortical level, during conditioning Som1 deactivated Pyr2 and activated Pyr1, causing LTP between MDT and Pyr1 and LTD between MDT and Pyr2 (Figure 2C). Overall, the increase in Hyp1 activity in VMHvl and the decrease in Pyr2 activity in mPFC recruited the dPag1 unit (Figure 2F). The repeated presentation of the conditioned stimuli (conspecific and context) without the defeat stimuli (lPBN and Som1 activation) slowly extinguished the activation of the dPAG (phase 3 of the protocol, Figure 2A). In this model, the extinction was driven by potentiation/depotentiation in mPFC, while the connections in the VMHvl remained stable (Figure 2C).

In classical fear conditioning, three classes of neurons have been described on the basis of their responsivity to the conditioned stimulus: the fear neurons are active after conditioning but not after extinction, the extinction neurons behave in the opposite way, and the persistent neurons are turned on after conditioning and remain responsive to the stimulus even after extinction (Repa et al., 2001; Milad and Quirk, 2002; Herry et al., 2008; Santini et al., 2008; Amano et al., 2010; An et al., 2012; Trouche et al., 2013). We looked for such populations in our model (Figure 2B). The unit Pyr1 in mPFC, that inhibits the activation of dPag1, behaved as a fear unit, while Pyr2 as an extinction unit (Figures 2B,D). We thus identified Pyr1 as a fear-ON population and Pyr2 as a fear-OFF population. Persistent neurons appeared in the VMHvl, represented by the unit Hyp1 (Figures 2B,E).

### 3.2. Alternative Model

**Figure 3:**
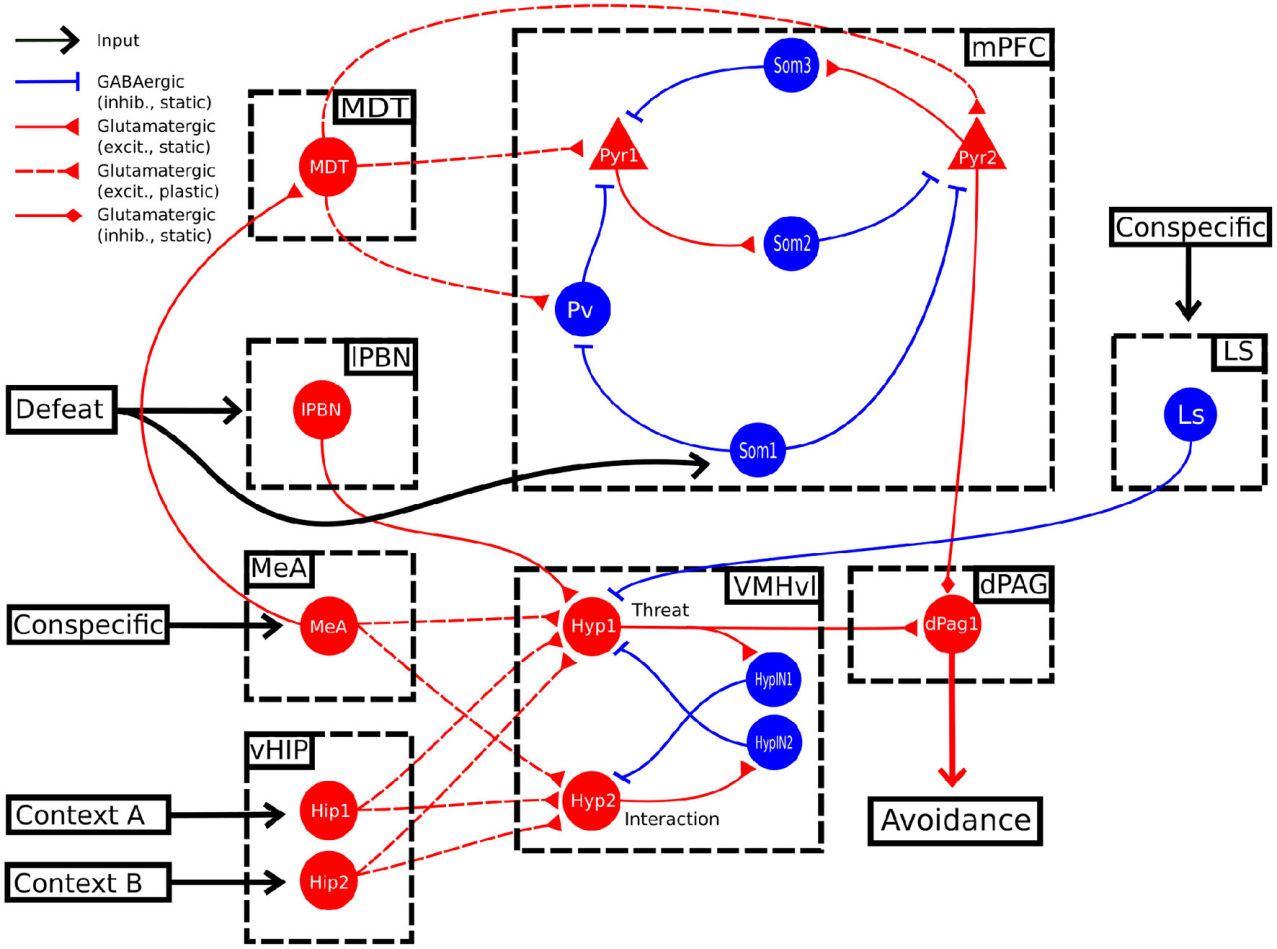
Alternative model. In this alternative model, the conspecific input also activates LS, which in turn sends an inhibitory projection to the unit Hyp1.

We explored in a second model (Figure 3) the possibility that the VMHvl is also involved in the extinction.

**Figure 4:**
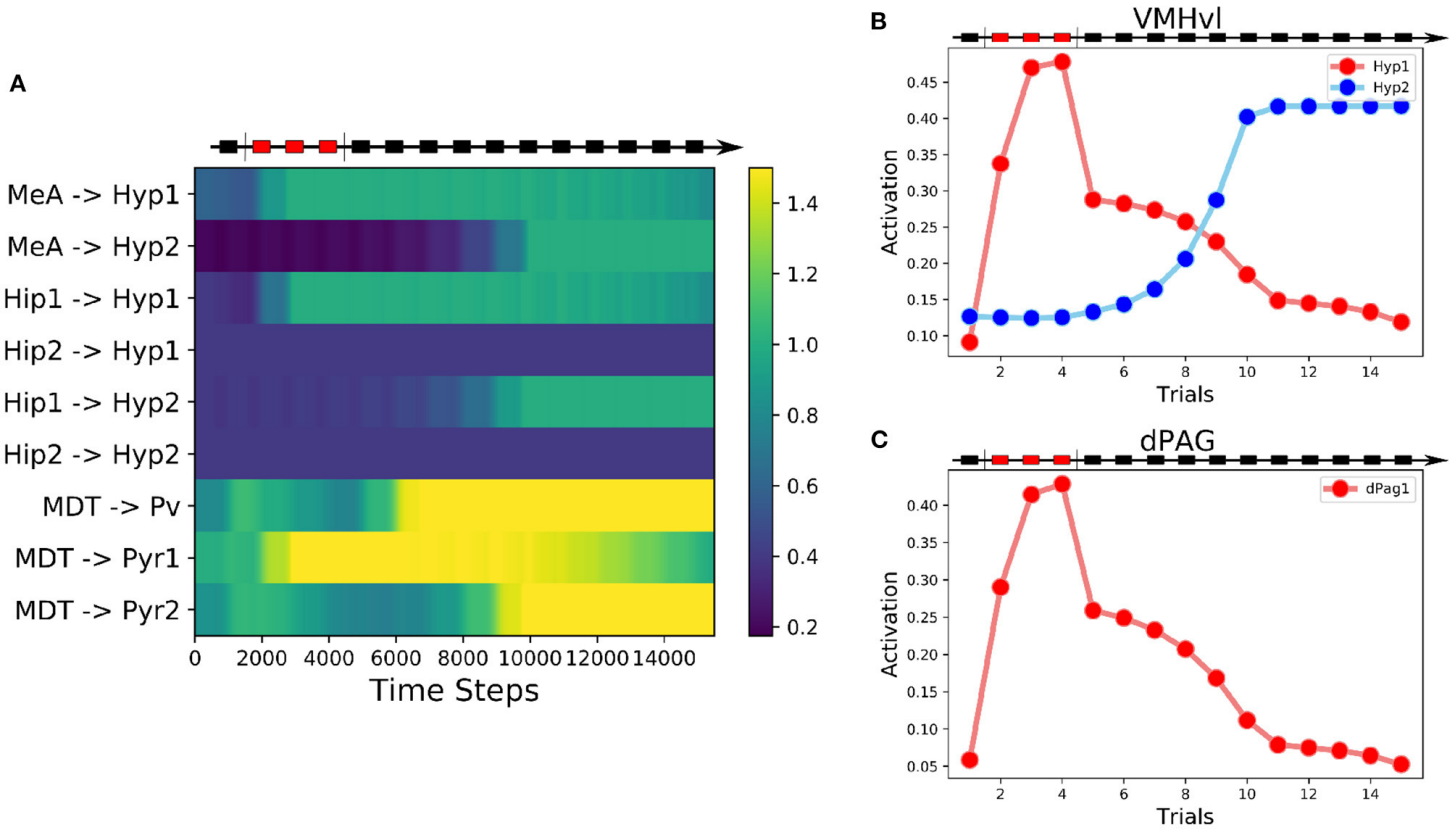
Behavior of the alternative model. **(A)** Heatmap of the weights of the model showing their evolution during the three-phases protocol. **(B)** Activation of the two excitatory units in the VMHvl. **(C)** Activation of the excitatory unit in the dPAG.

In this model, the Hyp1 unit must receive inhibitory input from the lateral septum (LS), representing a biasing signal elicited by interaction with a conspecific (Figure 3). As expected, during the conditioning trials the excitatory connections reaching Hyp1 were subjected to LTP (Figure 4A), increasing their activity and promoting the downstream activation of dPag1 (Figures 4B,C). As the extinction trial progressed, we observed the depotentiation of the connections reaching Hyp1 and the potentiation of those reaching Hyp2, indicating that the extinction progress is occurring also inside the VMHvl (Figures 4A,B). As a result, the activity of dPag1 diminishes during extinction (Figure 4C).

We modulated the output of the LS to the VMHvl, in order to simulate an experiment of LS inhibition during extinction. We observed that, compared to the control condition (Supplementary Figures S1A, S1B), a reduction of LS output of 30% slows down extinction (Supplementary Figures S1C, S1D), and a reduction of 38% completely abolishes it (Supplementary Figures S1E, S1F).

### 3.3. Reproduction of Key Target Experiments

Given that the alternative models exhibited a behavior very similar to the main model, except for the presence or absence of the persistent neurons in the VMHvl, we continued all the following simulations using the latter model (Figure 1). To validate the model, we verified if it could qualitatively fit some relevant experiments from literature (see Supplementary Methods for the detailed protocol of experiment reproduction and Supplementary Table S3).

**Figure 5:**
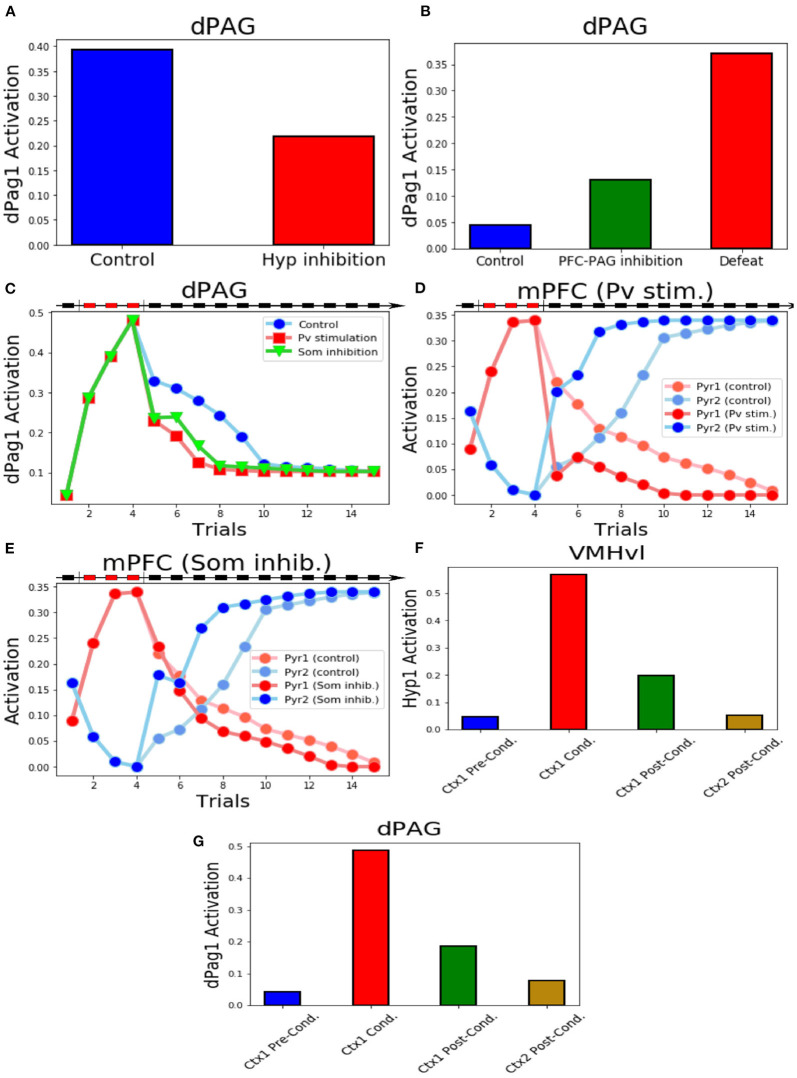
Key experiments reproduced by the model. **(A)** Inhibition of the excitatory units in VMHvl reduces the activity of dPag1. **(B)** Inhibition of the output of Pyr2 induces the activation of dPag1 in the system exposed to the conspecific and context input, even if it was never applied a protocol of conditioning. We show, for comparison, the dPag1 activation in a system subjected to the defeat protocol, where the unit Pyr2 is not manipulated. **(C)** The activation of Pv or the inhibition of Som1, Som2, and Som3 during the first trial of extinction, respectively, slow down or fasten the extinction process. **(D)** Behaviour of the excitatory units of the mPFC when Pv is stimulated during the first trial of extinction and respective controls **(E)** Behaviour of the excitatory units of the mPFC when Som1, Som2 and Som3 are inhibited during the first trial of extinction and respective controls. **(F,G)** Activation of the VMHvl unit Hyp1. **(F)** and the dPag1 **(G)** in context 1 before, during and after conditioning and in context 2 after conditioning.

First, we reproduced the data obtained by Silva et al. (2013) who found that pharmacogenetic inhibition of the VMHvl results in a significant decrease of the time spent in defensive postures during the exposure to an aggressive conspecific. In particular, we verified that blockage of the activity of all populations in VMHvl after social fear conditioning reduces the dPAG activation induced by the conspecific exposure (Figure 5A).

Franklin et al. (2017) demonstrated that the selective inhibition of the prefrontal projections from the layer 5 to the dPAG mimicked social avoidance in mice that had not experienced previous defeat. In the model, when the system receives the inputs representing the conspecific and the context, if the output of Pyr2 is prevented to reach dPAG we observe a substantial activity in the dPag1 population (Figure 5B). Indeed, the dPag1 population is under the control of both the excitatory input from Hyp1 and the inhibitory input from Pyr2 (Figure 1). In the absence of Pyr2, the excitatory input prevails and dPag1 becomes active (Supplementary Figure S2).

The experiments of Xu et al. (2019) provide insights about the functions of some populations of mPFC neurons in social fear conditioning. The model reproduces two key experiments of the authors, namely the stimulation of parvalbumin+ interneurons and the inhibition of the somatostatin+ interneurons after the three trials of social defeat. We observed that the activation of the Pv unit or the inhibition of the Som1, Som2, and Som3 units reduced the dPag1 activation induced by the defeat protocol (Figure 5C). We also observed a phenomenon that has not been investigated by Xu et al. (2019): the manipulation of the mPFC interneurons during the first extinction trial accelerated the subsequent acquisition of fear extinction, which thus was completed three trials before the control (Figure 5C). In particular, both the Pv stimulation and the inhibition of the Som units facilitated the potentiation of the connection between MDT and Pyr2 and the depotentiation of the connection between MDT and Pyr1 (Supplementary Figures S3A, S3B). This led to a more powerful activation of the Pyr2 fear-OFF population, compared to the non manipulated control, and to a consequent deactivation of the Pyr1 fear-ON population (Figures 5D,E).

Krzywkowski et al. (2020) studied the activation of different VMHvl populations during the aversive social encounter and when the mice were re-exposed to the same context without the opponent. Their Defeat+ population corresponds to our threat unit Hyp1, which is active during, but not before, conditioning (Figure 5F; see also Figure 2B). Re-exposure to the same context of the defeat, implemented through the activation of the Hip1 input without the conspecific or defeat inputs (MeA, lPBN, Som1 populations remained silent), recruited Hyp1 similarly to what done by the defeat. Conversely, exposure to a different context (Hip2) did not induce any Hyp1 activity (Figure 5F). This reproduces the findings of Krzywkowski et al. (2020) and implies that the connection between Hip1 and Hyp1 populations encodes the memory related to the contextual social fear conditioning. Indeed, we observed that in context 1 (Hip1 active), the activation of Hyp1 is able to drive dPag1 activation, even in the absence of the conspecific (Figure 5G).

### 3.4. Predictions

**Figure 6:**
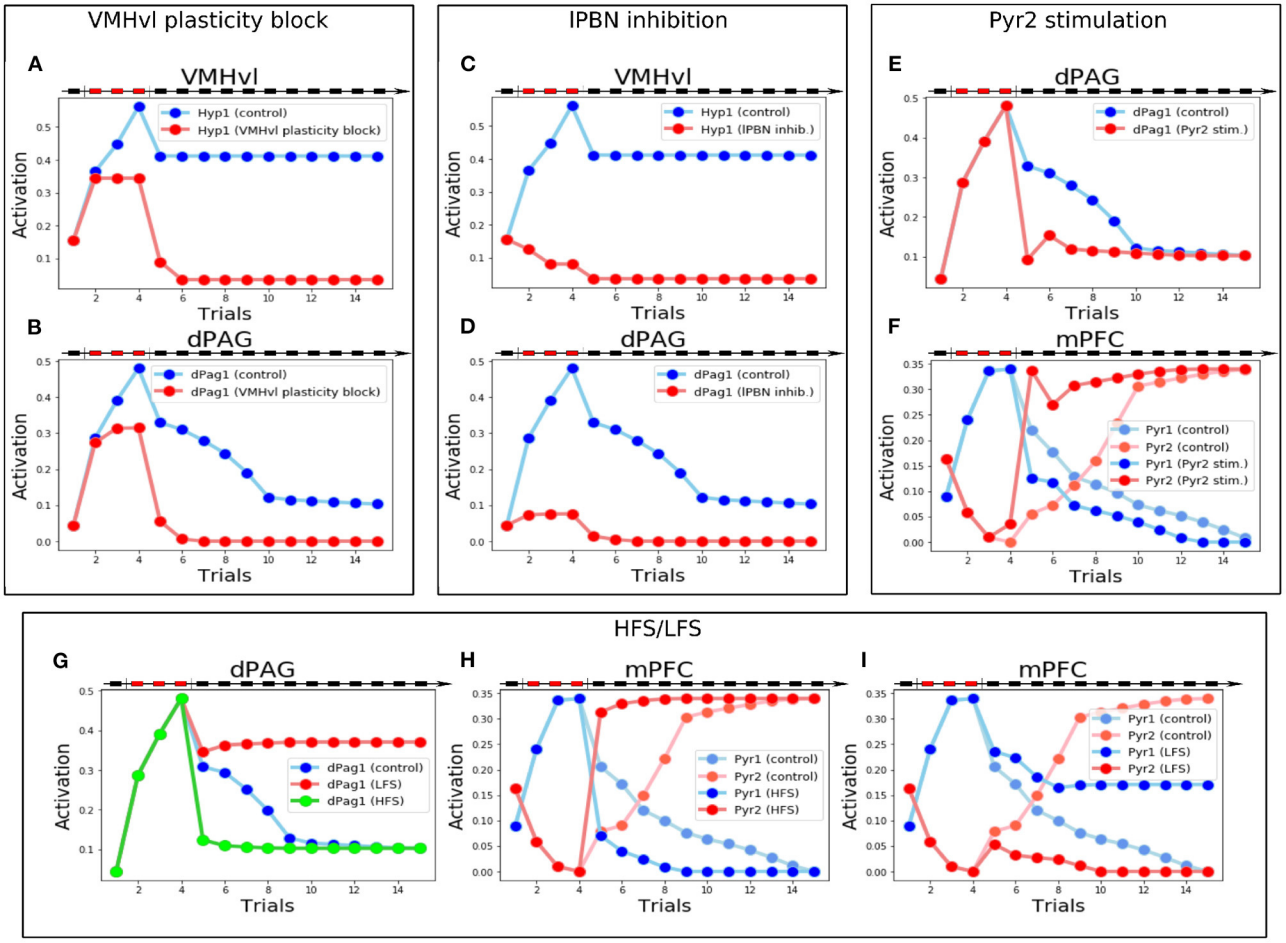
Manipulations of the model that generate testable predictions. **(A,B)** Activity of Hyp1 **(A)** and dPag1 **(B)**: with respect to the control, when the VMHvl plasticity is blocked during conditioning the memory of conditioning is fully lost. **(C,D)** Activity of Hyp1 **(C)** and dPag1 **(D)**: with respect to the control, when the lPBN activity is blocked during conditioning Hyp1 remains silent during the defeat and the extinction phases and dPag1 does not exhibit conditioning. **(E,F)** Stimulation of Pyr2 during the first trial of extinction: compared to the control, this changes the plasticity and activations in the mPFC **(E)** resulting in a fast deactivation of dPAG **(E)** and extinction. **(G–I)** Effects of MDT stimulation through a protocol mimicking HFS or LFS: with respect to the control, the dPag1 undergoes, respectively, a rapid drop or a lack of the decrease exhibited by the control **(G)**; in the case of HFS, after conditioning the mPFC Pyr1 activity decreases faster and Pyr2 activity increases faster than the control **(H)**; in the case of LFS, the mPFC Pyr1 does not decrease completely with extinction, and Pyr2 remains inhibited **(H)**.

The manipulations of the model that simulated plausible but never attempted *in vivo* experiments allowed us to make four specific testable predictions. First, we blocked synaptic plasticity inside the VMHvl area during the conditioning trials, in order to simulate an artificial impairment of LTP. As shown in Figures 6A,B, during the conditioning trials the Hyp1 and dPag1 units increase their excitation (although to a lesser extent than controls). However, their activation drastically decreases during the first trial of extinction (trial 5) and remains low in the last trials, thus indicating that the plasticity in VMHvl is mandatory for the acquisition of social fear memory.

The second prediction regards the role of the lPBN. We blocked its activity during the conditioning trials to simulate a pharmacological or optogenetic inhibition. Without the upstream input from lPBN, the hypothalamic fear population Hyp1 remains silent during the defeat and the extinction phases (Figure 6C). As a consequence, the connections between the input areas (MeA and Hip) and the VMHvl do not undergo synaptic plasticity (Supplementary Figure S4A) and dPag1, despite being slightly stimulated during the conditioning trials due to the weakening of the prefrontal inhibition (Supplementary Figure S4B), drastically decreases its levels of activation during extinction, indicating that the conditioning process is strongly impaired (Figure 6D).

The third prediction concerns what happens if the prefrontal Pyr2 population projecting to dPAG1 is stimulated after social conditioning. This corresponds to an experiment of pharmacological or optogenetic stimulation, executed during the first trial of extinction, of prefrontal neurons appropriately isolated from other PFC pyramidal neurons through retrograde labeling of the dPAG neurons. As shown in Figure 6E, the Pyr2 stimulation during the first extinction trial induces a rapid decrease of the levels of dPag1 activation compared to the control, which results in a one-trial extinction. This is due to the fact that in the mPFC (Figure 6F and Supplementary Figure S5) the strong activation of Pyr2 in the first extinction trial shuts down the competing Pyr1 population. The effect is an enhancement of LTP of the connection MDT-Pyr2 and the LTD of the connection MDT-Pyr1. This rapid reorganization in the mPFC circuit results in the dPag1 inhibition observed in Figure 6E.

The last prediction is linked to the results of Herry and Garcia (2002), who showed in *in vivo* experiments with mice undergoing auditory fear conditioning that the manipulation of the MDT-mPFC connections influences extinction. In particular, we investigated whether a similar outcome could be predicted for social fear conditioning. We, thus, exposed the model to a protocol that mimics the effect of either high-frequency stimulation (HFS) or low-frequency stimulation (LFS) of the MDT, administrated after conditioning (see Supplementary Methods for the details), to observe the effect on extinction. As shown in Figure 6G, HFS and LFS, respectively, accelerate or completely impair extinction. The cause of this is that HFS induces the potentiation of the MDT-Pyr2 connection. The simultaneous potentiation of the MDT-Pv connection (see Supplementary Materials), combined with the competition between Pyr1 and Pyr2, shuts down Pyr1 (Figure 6H) and this leads to a depotentiation of MDT-Pyr1. As a result, extinction is accelerated. LFS has the opposite effect: it induces a depotentiation of the MDT-Pyr2 and the MDT-Pv connections and, because of the resulting reduced inhibition on Pyr1, a potentiation of the MDT-Pyr1 connection (Supplementary Figure S7D). This results in an increase of the Pyr1 activity compared to the control, and in a full inhibition of the Pyr2 activity (Figure 6I). As a result, extinction is impaired.

### 3.5. Sensitivity Analysis

In order to examine the robustness of the model, we tested how changing the weights impacts on the reproduction of the conditioning, extinction and of the key target experiments (see Supplementary Methods, Supplementary Figure S6 and Supplementary Table S4). The model is very robust to the perturbation of almost all parameters. Notable exceptions are the weight of the connections between MDT and Pyr1 and between lPBN to Hyp1, that can be changed, respectively, in a range of 30 and 55%. This is reasonable, being these, respectively, the connection that conveys the defeat to the VMHvl and the connection mainly responsible for the reorganization of the mPFC after the conditioning. Finally, we observed that the HypIN1 neuron and the connection from Som1 to Pyr2 in mPFC can be eliminated from the model without affecting the outcome of the simulations.

## 4. Discussion

### 4.1. Contribution of the Model

In the field of Pavlovian cued and contextual fear conditioning, theoretical models have played an important role to integrate information and offer new perspectives on the experimental findings. This effort has produced novel predictions, has highlighted the knowledge gaps, and has driven further experimental research that has been used to build more sophisticated and biologically grounded models (Burgos and Murillo-Rodŕıguez, 2007; Mannella et al., 2008; Li et al., 2009, 2016; Krasne et al., 2011; Anastasio, 2013; John et al., 2013; Moustafa et al., 2013; Carrere and Alexandre, 2015; Kim et al., 2016; Nair et al., 2016; Oliva et al., 2018; Bennett et al., 2019; Mattera et al., 2020). We believe that social fear research could also benefit from this approach. Here, we have thus proposed a computational model of social avoidance that, to the best of our knowledge, is the first to address this complex phenomenon. A strong advantage of the model is that it ties together fragmented information regarding different brain areas involved in conditioning and extinction of social avoidance. In particular, social avoidance is mainly investigated with a focus on the VMHvl (Silva et al., 2013; Sakurai et al., 2016; Wang et al., 2019; Krzywkowski et al., 2020) or the mPFC (Franklin et al., 2017; Xu et al., 2019). However, how these structures interact and contribute to the phenomenon is a problem that has not yet been addressed experimentally.

In the proposed model, the threat neural population Hyp1 in VMHvl represents the Defeat+ population of Krzywkowski et al. (2020), shown to be activated by aversive social encounter with a conspecific. The activation of this population drives the firing of the dPAG neural population promoting the behavioral manifestations of fear (Figures 1, 2E,F). In agreement with this, it has been shown that the optogenetic activation of social fear neurons in VMHvl, marked by cFos expression after defeat, is sufficient to induce fear in mice (Sakurai et al., 2016). On the other hand, if these neurons are inhibited during an encounter with an aggressive conspecific, the defensive behavior is reduced (Silva et al. 2013; Figure 5A). The model proposes that the mPFC acts as a brake on subcortical structures, in particular by dampening the dPAG activation and thus preventing social avoidance expression. As shown by Franklin et al. (2017), and reproduced by the model (Figure 5B), the silencing of the connection between the mPFC and the dPAG induces fear in the undefeated mice. This fits with the finding for which a lesion of the mPFC reduces social interaction (Murray et al., 2015). In the model depicted in Figures 1, 3, the output unit dPag1 receives two distinct types of signals: the excitatory Hyp1 signal from the VMHvl and the inhibitory Pyr2 signal from the mPFC. In the baseline condition, before conditioning the presence of a conspecific activates only loosely the Hyp1 unit (Figure 2B), and the concomitant activation of Pyr2 is enough to turn off dPag1. In order to elicit social avoidance, the Hyp1 population must be more strongly activated by a conspecific [as artificially done by Sakurai et al. (2016)] or the Pyr2 population must be less responsive [as artificially done by Murray et al. (2015), Franklin et al. (2017)]. Social defeat triggers a reorganization of the synaptic weights in VMHvl and in the mPFC such that both conditions are met (Figure 2C). During extinction, the relative levels of the mPFC Pyr2, but not Hyp1, return to the naive condition the main model (Figure 2). Instead, the excitation of both Hyp1 and Pyr2 are restored in the alternative model (Figure 4A).

On the basis of the overall picture emerging from these results, the model suggests that a possible rescue strategy for the social avoidance disease (SAD) is to restore, or potentiate, the brake function of the mPFC on the subcortical circuit formed by the threat population Hyp1 of the VMHvl and the dPAG. In support of this view, imaging studies have shown an altered activation of the mPFC in SAD patients (Labuschagne et al., 2012; Stein, 2015). Moreover, remarkably in SAD the functional connectivity between the mPFC and subcortical structures is significantly decreased (Prater et al., 2013; Gold et al., 2016). This pathological condition is reproduced in the murine model of sub-chronic social defeat, where the local field potential coherence between the mPFC and dPAG is reduced (Franklin et al., 2017). Conversely, symptom improvement after cognitive-behavioral therapy (CBT) is predicted by an increase of mPFC-subcortical connectivity (Klumpp et al., 2014).

From a theoretical perspective, what is the need for a social animal to have both an “accelerator” (VMHvl) and a “brake” (mPFC) of the social avoidance? The prefrontal cortex is an area that underlies executive functions (working memory, inhibition, flexibility) to support goal-directed behavior (Uylings et al., 2003; Kesner and Churchwell, 2011; Diamond, 2013). It has been recently highlighted that the prefrontal regulation of the basolateral amygdala in auditory and contextual fear conditioning should be considered in this framework (Gonzalez and Fanselow, 2020). In this view, it seems plausible that, when some particular goals are pursued, an animal could get advantaged to override a subcortical impulse to flight through the inhibitory cortical control of sub-cortical areas.

Besides executive functions, another factor that could influence social avoidance is social rank. Notably, social rank is encoded in the mPFC and shares some pathways with those of social fear. In particular, the strength of the connection between the MDT and the mPFC, and the excitability in layer 5 pyramidal neurons, determine the animal hierarchical status (Wang et al., 2011; Zhou et al., 2017). As expected from this, it has been observed that the animal rank predicts the susceptibility to a social defeat paradigm (Larrieu et al., 2017).

### 4.2. Anatomical and Functional Considerations

In the construction of the model mPFC, we derived the neural populations and the connections between them from both experimental findings on the activation/deactivation of different neurons during social conditioning, and from some hypotheses formulated on the basis of the analysis of the literature (see Supplementary Table S1). Here we thus speculate on the possible localization of these populations in the mPFC anatomy.

We propose that the brain correspondent of the model neural population Pyr2 is located in layer 5, having been shown that the descending projections from mPFC to dPAG originate from it (Franklin et al., 2017). The mPFC lacks a thalamorecipient layer and the MDT input reaches both layer 5 and layer 2/3 (Collins et al., 2018). It is thus possible MDT reaches Pyr2 through a direct connection. The location of the Pyr1 population is more difficult to infer. These neurons are specifically activated by social fear conditioning, as shown with c-Fos labeling and calcium imaging (Xu et al., 2019). Remarkably, Xu et al. (2019) reported a significant activation of c-Fos in prelimbic, but not infralimbic cortex. On the other hand, Hinwood et al. (2011) previously showed that a protocol of repeated social defeat recruits ΔFosB positive neurons in layer 2/3 of the infralimbic cortex. Although the protocols are different, this suggests the possibility that the neurons described by Xu et al. (2019) belong to the higher layers of the mPFC. Unfortunately, there is paucity of experimental data on the involvement of prelimbic and infralimbic cortex in social avoidance after a sub-chronic social defeat or a social fear conditioning protocol. Future work could aim to investigate if the dynamics of the neural populations reproduced in the mPFC of our model could be related to the behaviour of prelimbic and infralimbic populations.

Following the aforementioned experimental findings, the parvalbumin+ population (represented in our model by the unit Pv, Figure 1), that after social fear conditioning becomes hyperactive and depresses the pyramidal neurons firing during conspecific encounters (Xu et al., 2019), is the one supporting feedforward inhibition in layer 2/3 after MDT stimulation (Delevich et al., 2015). We could also try to locate the interneuronal somatostatin+ populations represented in our model by Som2 and Som3. Som2, which in our model is activated by Pyr1 and inhibits Pyr2, could represent a translaminar inhibitory circuit from layer 2/3 to layer 5. Optogenetic mapping suggests that somatostatin+ interneurons that receive from layer 2/3 and project to layer 5 are located within layer 5 (Naka and Adesnik, 2016). On the other hand, cases have been described of somatostatin+ cells from layer 5 that project back to pyramidal neurons in layer 2/3 (Kapfer et al., 2007; Nigro et al., 2018), as could be for the population represented by our Som3 unit.

One of the main mechanisms of social avoidance that still lacks an explanation supported by empirical evidence is the “defeat signal” that triggers the functional reorganization of the mPFC. We observed (see Materials and Methods) that the mPFC disinhibitory circuit comprising somatostatin+ and parvalbumin+ interneurons upstream of the pyramidal neurons is shared by auditory and social fear conditioning (Courtin et al., 2014; Xu et al., 2019; Cummings and Clem, 2020). A similar disinhibitory mechanism is present in the auditory cortex, where layer 1 interneurons negatively regulate layer 2/3 parvalbumin+ interneurons, which in turn inhibit excitatory layer 2/3 neurons (Letzkus et al., 2011). Auditory fear conditioning requires that the auditory cortex is disinhibited through the activation of layer 1 interneurons at the moment of fear learning. The key element that recruits layer 1 interneurons is the nicotine acetylcholine (ACh) receptors (Letzkus et al., 2011). It has been shown that pairing a tone with the stimulation of the nucleus basalis, that releases ACh to the cortex, causes an unbalanced excitation/inhibition through the induction of synaptic plasticity (Froemke et al., 2007). Thus, in certain conditions, ACh acts as a negative reward signal that remodels cortical synapses, as also observed using a go/no-go paradigm (Hangya et al., 2015). In the mPFC, ACh from the nucleus basalis mainly targets somatostatin+ interneurons (Sun et al., 2019). On the basis of this evidence, and in line with an idea recently discussed in the literature (Wang et al., 2020), we suggest that the defeat signal recruiting somatostatin+ interneurons in mPFC (Som 1 in our model, see Figure 1) during social fear conditioning could be represented by ACh. We propose that a possible experiment usable to investigate a coincident activation of the central cholinergic neurons during the social fear protocol could be the *in vivo* electrophysiological recordings in the nucleus basalis, paired with an identification of the ACh-releasing neurons through optogenetic tagging (Lima et al., 2009; Hangya et al., 2015). If an activation of cholinergic neurons is recorded, a direct way to demonstrate the role of ACh in the mPFC in fear conditioning could be the *in loco* administration of antagonists of ACh receptors (Gu et al., 2020). Finally, cell specific knockout of the receptors (Hernandez et al., 2014; Gu et al., 2020) could be used to locate the subtype of neurons on which ACh acts to produce the fear conditioning.

In the alternative model (Figure 3) we proposed the existence of an inhibitory input on the threat neurons in the VMHvl. When this modification is inserted in the model, the persistent population in VMHvl is substituted by an extinction population. We speculate that this possible inhibitory afferent is represented by the LS, a key area involved in social interaction and social fear pathways. Indeed, LS sends inhibitory projections to the VMHvl to control aggressive behaviors against conspecifics and, when optogenetically stimulated, promotes social investigation (Wong et al., 2016). Moreover, oxytocin (OXT), which has pro-social effects and has been proposed as a therapy for SAD (Guastella et al., 2009; Labuschagne et al., 2010), when directly administrated to the LS of mice facilitates social fear extinction (Zoicas et al., 2014; Menon et al., 2018). This suggests the idea that neural projections from the main oxytocinergic hypothalamic nuclei, such as the paraventricular nucleus and particularly the supraoptic nucleus, are important upstream regulators of the levels of inhibition provided by the LS to the VMHvl. It is worth noting that social fear conditioning induces a reduction of the levels of oxytocin release inside the LS, with a mechanism still to be explored (Zoicas et al., 2014). This is possibly due to the alterations induced by social fear conditioning inside the main oxytocinergic nuclei. Indeed, it has been observed that stress has a detrimental impact on the PVT, inducing a reduction of the expression of serotonin 5-HT1A receptors (Florez et al., 2017). Future studies are required to elucidate the involvement of this pathway on social fear conditioning and extinction, especially in relation to the presence or absence of persistent neurons in VMHvl. As we observed in Supplementary Figure S1, in the alternative model the inhibition of LS slows down extinction and, if sufficiently high, inhibits it. This experiment, once performed in mice, would allow the discrimination which model is more accurate in the description of murine social avoidance pathways.

### 4.3. Predictions of the Model

Our model produced various predictions (Table 1), some derived from specific manipulations of the model aimed to simulate possible new experiments and others deduced from the reproduction of data already present in the literature.

**Table 1:** Testable predictions of the model.

**Prediction**	**Possible test strategy**
Presence of fear and extinction neurons in mPFC; presence of persistent neurons in VMHvl	*In vivo* recordings in mPFC and VMHvl during a conspecific exposure, done before conditioning, after conditioning and after extinction
Blocking the plasticity in VMHvl does not alter the social fear responses but impairs the formation of the fear memory	Stereotactic injection of AP5 or other plasticity inhibitors in VMHvl before social fear conditioning
lPBN nucleus conveys the nociceptive information necessary to the social conditioning to the VMHvl	Stereotactic injection of muscimol in lPBN before conditioning
Layer 5 mPFC pyramidal neurons stimulation during a social encounter boosts the extinction of social fear	Administration of drugs that enhance the excitability of layer 5 neurons, like LSD, before extinction
HFS and LFS of MDT, respectively, increase or completely abolish extinction	*In vivo* HFS of LFS of MDT with electrodes before extinction

We observed that, similarly to what happens in auditory and contextual fear conditioning, the model exhibits the three classes of fear, extinction, and persistent neural populations. In particular, on the basis of our simulations, we expect to find fear and extinction neurons in the mPFC, corresponding to, respectively, the neural populations Pyr1 and Pyr2 (Figure 2B). Pyr1 corresponds to the population of neurons activated by the conspecific exposure in the socially conditioned mice (Xu et al., 2019). Unfortunately, the authors of the research did not investigate the effect of the extinction on the firing of this population, and it is thus not possible to label them as fear or persistent neurons. On the other hand, the simulations predict that VMHvl contains persistent neurons (Figure 2B), unless a biasing signal inhibits the threat population during social encounters, such as LS inhibition in the alternative model (Figure 2). Persistent neurons have an important implication in the context of SAD. Indeed, it has been observed that the existence of these neurons in the amygdala after auditory fear conditioning prevents the return to a pre-conditioning state even after extinction. For this reason, the fear memory can be easily reinstated (Maren and Holmes, 2016). Moreover, if the subcortical areas, but not the mPFC, contain persistent neurons, this would imply that the extinction is entirely entrusted to the cortex, that thanks to its flexibility is able to return to the naive condition.

After blocking the plasticity of the VMHvl during the conditioning procedure the model is still able to acutely evoke the avoidance response. However, the acquisition of social avoidance is drastically impaired and the model fails to elicit the fear response in the following trials (Figures 6A,B). This result has analogies with the results of the experiments of Silva et al. (2016b), where it was seen that silencing the dorsomedial part of the VMH is sufficient to impair the acquisition of predatory fear memory. We speculate that the same process occurs inside the VMHvl during conditioning and that the plasticity taking place in this structure is fundamental for the integration of contextual, nociceptive, and social information, and for the acquisition of social avoidance.

An important afferent of the model is the lPBN, which delivers pain signals to different subcortical structures to drive appropriate responses (Chiang et al., 2019, 2020). In particular, the activation of the projections to the VMHvl induces escape (Chiang et al., 2020). Moreover, lPBN input to the amygdala is necessary and sufficient to induce fear memories (Sato et al., 2015; Chiang et al., 2020). We hypothesized that the unconditioned signal necessary for the conditioning of the subcortical compartment arises from this area. For this reason, we predict that its inhibition would result in a failed conditioning (Figures 6C,D).

When we stimulated the unit Pyr2 during the first trial of extinction, we observed a fast one-shot abolishment of social avoidance (Figure 6E). This is especially interesting in the context of a possible pharmacological treatment of SAD. In the light of the aforementioned speculations regarding the localization of the Pyr2 population in the mPFC, we suggest that a therapy of stimulation of the layer 5 pyramidal neurons projecting to the dPAG during the exposure to the fearful stimuli could induce a fast therapeutic recovery. In this perspective, a possible pharmacologic agent would be LSD, an agonist of 5-HT2A receptors (Nichols, 2004). Indeed the activation of these receptors enhances glutamatergic currents in the apical dendrites of layer 5 pyramidal neurons in mPFC (Aghajanian and Marek, 1997, 1999). In addition, LSD has been recently shown to promote social behavior in rodents through the activation of the prefrontal excitatory neurons. Optogenetic inhibition of the mPFC, on the other hand, not only blocks the pro-social effect of LSD, but also induces social avoidance (De Gregorio et al., 2021; this is in line with the simulation shown in Figure 2B, where we blocked the output of the mPFC). It is worth noting that an LSD-induced enhancement of sociability has also been reported in humans (Dolder et al., 2016; Duerler et al., 2020). Remarkably, LSD has never been investigated in defeated mice but our simulations and our theoretical framework support the hypothesis of a beneficial effect on social avoidance.

The last prediction regards how an MDT stimulation inducing plasticity to the connections with the mPFC influence extinction. We were interested in this simulation for 2 reasons. First, this manipulation determines the success of extinction to auditory conditioning (Herry and Garcia, 2002). We reasoned that a similar outcome would suggest that auditory and social fear share some pathways or mechanisms at the level of mPFC. Second, the strength of the MDT-mPFC synapse determines the social rank of the rodents and influences the winning individual in the tube test (Zhou et al., 2017). The outcome of our simulations (Figures 6G–I) was very similar to those obtained by Herry and Garcia (2002), where HFS and LFS, respectively, increase or completely abolish extinction. Unfortunately, the influence of rank on social fear is still poorly investigated (Larrieu et al., 2017), but our results raise the possibility that an increase in the social rank scale would have a positive effect on the social fear extinction.

### 4.4. Limitations of the Model

We now discuss some limitations of the model that should be addressed in future work. The first limitation regards the connectivity of the mPFC with other structures beyond dPAG. For example, layer 2/3 excitatory neurons (possibly our Pyr1 population) send projections to the Nucleus Accumbens (NAcc; Franklin et al. 2017) and this structure has been shown to be involved in the expression, but not the acquisition, of social fear (Luckett et al., 2012). This suggests that NAcc and its downstream areas are involved in the fear-promoting pathway of the Pyr1 population. Besides NAcc, it has been recently shown that the pathway connecting the mPFC to the paraventricular nucleus of the thalamus (PVT) is also part of the sociability circuit and that the inhibition of the mPFC to PVT pathway leads to a reduced social preference (Yamamuro et al., 2020). Here, we focused on a minimal circuit able to explain the principal experimental findings and to produce predictions without constructing an overly complex architecture. For the same reason, we have not taken into account other neural structures involved in parallel or complementary circuits of social fear behavior such as the bed nucleus of the stria terminalis (Clauss et al., 2019) and the dorsal pre-mammillary nucleus (Motta et al., 2009). These structures were not necessary to explain the experiments targeted here but they should be taken into account in future models.

Another limitation of this work regards the kind of modeling approach employed in this work. In particular, the firing rate neural model is capable to simulate the long-range projections and the excitatory/inhibitory relationship between the different brain areas, but says little about their internal computation and cannot reproduce some complex phenomena. For instance, a recent work of the group of Xu (Liu et al., 2020) revealed unexpected complexity in the social control exerted by the mPFC. In particular, the authors observed that activation of parvalbumin+, but also somatostatin+ interneurons at the gamma frequency, results in a pro-social effect. On the other hand, a nonrhythmic activation of somatostatin+ interneurons has the opposite outcome. Investigating these phenomena would give a more complete understanding of the mPFC control of social fear, but would also require spiking neural networks to simulate brain oscillations (e.g., Wei et al. 2016; Capone et al. 2019).

Notwithstanding these needed developments, as happened for the classical fear conditioning studies centered on the amygdala, the results presented here should have demonstrated the utility of studying social avoidance through top-down models. These models are indeed capable of systematising and conceptualising the experimental findings on the involved neural areas and plasticity mechanisms and also of paving the way to additional studies using more detailed bottom-up models, as it is done in the study of auditory fear conditioning (Nair et al., 2016).

## Data Availability Statement

The original contributions presented in the study are included in the article/Supplementary Material, further inquiries can be directed to the corresponding author/s.

## Author Contributions

VA, AM, and GB: conceived the model, conducted the simulation, analyzed the results, and wrote and reviewed this article. All authors contributed to the article and approved the submitted version.

## Funding

This project has received funding from the European Union's Horizon 2020 Research and Innovation Programme under Grant Agreement No. 713010 (GOAL-Robots–Goal-based Open-ended Autonomous Learning Robots).

## Conflict of Interest

The authors declare that the research was conducted in the absence of any commercial or financial relationships that could be construed as a potential conflict of interest.

## Publisher's Note

All claims expressed in this article are solely those of the authors and do not necessarily represent those of their affiliated organizations, or those of the publisher, the editors and the reviewers. Any product that may be evaluated in this article, or claim that may be made by its manufacturer, is not guaranteed or endorsed by the publisher.
