# Supplementary material for: Neural Circuits Underlying Social Fear in Rodents: An Integrative Computational Model
Source: Front Syst Neurosci. 2022 Mar 8;16:841085. doi: 10.3389/fnsys.2022.841085 (PMC8957808; doi:10.3389/fnsys.2022.841085)
Supplement: Supplementary file 1 [file Data_Sheet_1.pdf]

frontiers

Supplementary Material

1 SUPPLEMENTARY METHODS

1.1 Simulation protocols for the key target experiments

We challenged the model to fit different experimental findings from the literature (Table S3). To this purpose, we tested the model with experimental protocols as similar as possible to those used in the target real experiments.

Silva et al. (2013) studied the effect of VMHvl inhibition on defensive behavior during conspecific aggression. To inhibit the VMHvl neurons they employed hM4D–clozapine-N-oxide, with hM4D virally expressed in approximately 40% of neurons. To mimic this experiment, we subjected the model to a single trial of conditioning while simultaneously reducing the activity of all the neural units of the VMHvl (Hyp1, Hyp2, HypIN1 and HypIN2) by 40%. As a control, the model was exposed to a single trial of conditioning without the inhibition of the VMHvl units.

Xu et al. (2019) showed that, after a protocol of fear conditioning, pharmacologically inhibiting somatostatin+ neurons or stimulating parvalbumin+ neurons by using respectively hM4D–clozapine-N-oxide or hM3D–clozapine-N-oxide in mPFC reduced social avoidance. To simulate these experiments, in the first trial of extinction we reduced the activity of the neural units Som1, Som2 and Som3 by 83.3% (the percentage of somatostatin+ cells targeted by Xu et al. (2019)) or stimulated with an external input the Pv population (to mimic the pharmacogenetic activation of parvalbumin+ neurons as in Xu et al. (2019)).

Franklin et al. (2017) demonstrated that selectively inhibiting the prefrontal projections from the mPFC to the dPAG, by using hM4D–clozapine-N-oxide, resulted in increased levels of social avoidance during the interaction with a conspecific. To simulate this experiment, we tested the model in a single interaction trial while simultaneously inhibiting the prefrontal unit Pyr1. Due to the fact that the authors did not report the exact infection efficiency, we arbitrarily used a inhibition of 83.3% (as in Xu et al. (2019)). We compared the results with those of the naive model and of the conditioned model after the three trials of the defeat protocol.

Krzywkowski et al. (2020) exposed the conditioned mice to the same context of social defeat, but without the aggressor mouse, and measured the activation of neurons in the VMHvl through *in vivo* calcium imaging. We exposed our model to one trial of baseline exploration of context 1 (Ctx1, represented by the activation of the input unit Hip1) followed by three trials of conditioning. After conditioning, we exposed the model either to the context associated with conditioning (Ctx1) or to a different context (Ctx2, represented by the activation of input unit Hip2) as a control.

While the previous manipulations were devised to reproduce the experimental findings, the following ones explored novel scenarios to obtain testable predictions with the model. The first manipulation consists of blocking the plasticity in VMHvl to study the effect on the conditioning process. To simulate this, we exposed the model to one trial of baseline interaction, three trials of conditioning with impaired plasticity of the hypothalamic units (Hyp1, Hyp2, HypIN1, HypIN2), followed by 11 trials of extinction.

We then investigated whether the inhibition of lPBN during the social defeat procedure impairs social fear expression during and after conditioning. We simulated this experiment by subjecting the model to one trial of baseline interaction, three trials of conditioning with lPBN inhibited, and 11 trials of extinction.

To confer realism to the manipulation, we reduced the lPBN activation of 80.0% during conditioning as this is a plausible percentage of cells inhibited in a real experiment Xu et al. (2019).

The third manipulation aimed to observe the effect on social avoidance of an external stimulation ($I = 0.4$) of the prefrontal projections to the dPAG after conditioning. The experiment was simulated by exposing the model to one trial of baseline interaction, three trials of conditioning, one trial of post-conditioning interaction where we stimulated with and external input the Pyr2 unit, and ten trials of extinction.

Finally, we investigated how the modulation of the activity of the thalamic input to mPFC through a protocol inducing long term depression (LTD) or potentiation (LTP) influences social fear. This manipulation takes inspiration from what done by Herry and Garcia (2002) for auditory fear conditioning, where low frequency (LFS, corresponding to 2 Hz) or high frequency stimulation (HFS, corresponding to 250 Hz) were applied to the MD to obtain respectively LTD or LTP in the MD-mPFC connection. To do this, first we found the presynaptic level of stimulation that could mimic the LFS and HFS protocols using a simplified system consisting of a single presynaptic and a single postsynaptic population (Figure S7A). On this basis, we observed that a stimulation of 0.1 for 15 trials corresponds to the LFS protocol inducing LTD in the postsynaptic neuron, and a stimulation of 1.0 for 15 trials corresponds to the HFS protocol inducing LTP (Figure S7B). We then applied these two manipulations to the whole model to confirm that they were capable of modifying the weights of the connections in the mPFC (Figure S7C and S7D).

1.2 Sensitivity analysis

We investigated the sensitivity of the model to the parameter changes through the *One-factor-At-a-Time procedure* Saltelli et al. (2008). The technique consists of changing the reference value of a single parameter at a time with 5% steps towards zero or +100%, while leaving the other parameters intact. The procedure ended and we moved to the next parameter if a) the model failed to reproduce an experiment (conditioning, extinction or one of the 5 key target experiment); or b) the parameter reached the value of zero (meaning that the connection could be removed without affecting the experiments) or the value of +100%.

2 SUPPLEMENTARY TABLES

Table S1. Parameters of the firing rate units and the plasticity.

**Parameter**	**Value**
Tau	100
Δt	1
I	1
α	0.01
θ mPFC	0.045
θ subcortical	0.2
Wmin	0.1
Wmax mPFC	1.0
Wmax subcortical	1.5

Table S2. Synaptic connections and weights. List of synaptic connections of the two different models, the references regarding the existence of these connections and the weights assigned through the manual searching procedure.

Connection	Reference	Weight	Weight (alternative model)	Plasticity
MDT to Pyr1	Collins et al. (2018)	1.0	1.0	Yes Herry and Garcia (2002); Zhou et al. (2017); Franklin et al. (2017)
MDT to Pyr2	Collins et al. (2018)	0.85	0.85	Yes Herry and Garcia (2002); Zhou et al. (2017); Franklin et al. (2017)
MDT to Pv	Delevich et al. (2015)	0.8	0.8	Yes Nelson et al. (2019)
Som1 to Pv	Naka and Adesnik (2016)	1.25	1.25	No
Som1 to Pyr2	Silberberg and Markram (2007); Naka and Adesnik (2016)	0.45	0.45	No
Pv to Pyr1	Naka and Adesnik (2016)	1.2	1.2	No
Pyr1 to Som2	Silberberg and Markram (2007); Naka and Adesnik (2016)	1.5	1.5	No

Som2 to Pyr2	Silberberg and Markram (2007); Naka and Adesnik (2016)	2.0	2.0	No
Pyr2 to Som3	Silberberg and Markram (2007); Naka and Adesnik (2016)	1.7	1.7	No
Som3 to Pyr1	Silberberg and Markram (2007); Naka and Adesnik (2016)	1.5	1.5	No
Pyr2 to Pag1	Franklin et al. (2017)	2.0	0.5	No
MeA to Hyp1	Nordman et al. (2020)	0.6	0.6	Yes Nordman et al. (2020)
MeA to Hyp2	Nordman et al. (2020)	0.2	0.2	Yes Nordman et al. (2020)
MeA to MDT	Krettek and Price (1977); Canteras et al. (1995); Mitchell and Chakraborty (2013)	2.45	2.45	No
Hyp1 to Pag1	Silva et al. (2013)	2.0	2.0	No

lPBN to Hyp1	Fulwiler and Saper (1984); Chiang et al. (2020)	1.0	1.0	No
Hip1 to Hyp1	Chang and Gean (2019)	0.4	0.4	Yes (Hypothesis)
Hip1 to Hyp2	Chang and Gean (2019)	0.4	0.4	Yes (Hypothesis)
Hip2 to Hyp1	Chang and Gean (2019)	0.4	0.4	Yes (Hypothesis)
Hip2 to Hyp2	Chang and Gean (2019)	0.4	0.4	Yes (Hypothesis)
Hyp1 to HypIN1	Hypothesis	0.3	0.3	No
Hyp2 to HypIN2	Hypothesis	1.0	2.0	No
HypIN1 to Hyp2	Hypothesis	0.3	0.3	No
HypIN2 to Hyp1	Hypothesis	1.0	2.0	No
Ls to Hyp1	Risold and Swanson (1997); Wong et al. (2016)	Absent	0.23	No

Table S3. Experiments used to validate the model, model predictions, and manipulations of the model performed to obtain the result/prediction.

Experiment	Result	Reference	Input	Manipulation
Inhibition of the VMHvl during social defeat	Reduced social fear	Silva et al. (2013)	MeA, Hip1, lPBN, Som1 (defeat)	Inhibition (Hyp1, Hyp2, HypIN1, HypIN2)

Inhibition of Som neurons after conditioning	Reduced social fear	Xu et al. (2019)	MeA, Hip1 (pre-defeat); MeA, Hip1, lPBN, Som1 (defeat); MeA, Hip1 (post-defeat)	Inhibition (Som1, Som2, Som3)
Stimulation of PV neurons after conditioning	Reduced social fear	Xu et al. (2019)	MeA, Hip1 (pre-defeat); MeA, Hip1, lPBN, Som1 (defeat); MeA, Hip1 (post-defeat)	Stimulation (Pv)
Inhibition of mPFC to dPAG projections	Enhanced social fear	Franklin et al. (2017)	MeA, Hip1 (pre-defeat); MeA, Hip1, lPBN, Som1 (defeat); MeA, Hip1 (post-defeat)	Inhibition (Pyr2)
Re-exposure to the social conditioning context	Recruitment of the same population activated during defeat in VMHvl	Krzywkowski et al. (2020)	Hip1 (pre-defeat); MeA, Hip1, lPBN, Som1 (defeat); Hip1 or Hip2 (post-defeat)	Activation of Hip1 but not MeA after conditioning
Inhibition of the plasticity in VMHvl	Impaired social fear conditioning	Prediction of the model	MeA, Hip1 (pre-defeat); MeA, Hip1, lPBN, Som1 (defeat); MeA, Hip1 (post-defeat)	Plasticity block (Hyp1, Hyp2)
Inhibition of lPBN activity	Impaired social fear expression and conditioning	Prediction of the model	MeA, Hip1 (pre-defeat); MeA, Hip1, lPBN, Som1 (defeat); MeA, Hip1 (post-defeat)	Inhibition (lPBN)
Stimulation of mPFC to dPAG projections	Reduced social fear	Prediction of the model	MeA, Hip1 (pre-defeat); MeA, Hip1, lPBN, Som1 (defeat); MeA, Hip1 (post-defeat)	Stimulation (Pyr2)
HFS of MDT to mPFC projections before extinction	Enhanced extinction	Prediction of the model	MeA, Hip1 (pre-defeat); MeA, Hip1, lPBN, Som1 (defeat); MeA, Hip1 (post-defeat)	Stimulation of 1.0 for 15 trials (MDT)
LFS of MDT to mPFC projections before extinction	Impaired extinction	Prediction of the model	MeA, Hip1 (pre-defeat); MeA, Hip1, lPBN, Som1 (defeat); MeA, Hip1 (post-defeat)	Stimulation of 0.1 for 15 trials (MDT)

Table S4. Results of the sensitivity analysis, with the maximum values of the decrements and increments that can be applied without losing one of the experiments.

Parameter	Decrements	Increments

Ls to Hyp1	-35%	+100%
HypIN2 to Hyp1	-25%	+100%
HypIN1 to Hyp2	-100%	+10%
Hyp2 to HypIN2	-25%	+100%
Hyp1 to HypIN1	-100%	+10%
Hip2 to Hyp2	-95%	+100%
Hip2 to Hyp1	-70%	+15%
Hip1 to Hyp2	-95%	+100%
Hip1 to Hyp1	-70%	+15%
lPBN to Hyp1	-30%	+25%
Hyp1 to Pag1	-95%	+100%
MeA to MDT	-5%	+95%
MeA to Hyp2	-95%	+100%
MeA to Hyp1	-25%	+100%
Pyr2 to Pag1	-95%	+100%
Som3 to Pyr1	-30%	+100%
Pyr2 to Som3	-30%	+100%
Som2 to Pyr2	-95%	+5%
Pyr1 to Som2	-95%	+5%
Pv to Pyr1	-10%	+100%
Som1 to Pyr2	-100%	+25%
Som1 to Pv	-75%	+100%
MDT to Pv	-10%	+55%
MDT to Pyr2	-0%	+55%
MDT to Pyr1	-25%	+5%

3 SUPPLEMENTARY FIGURES

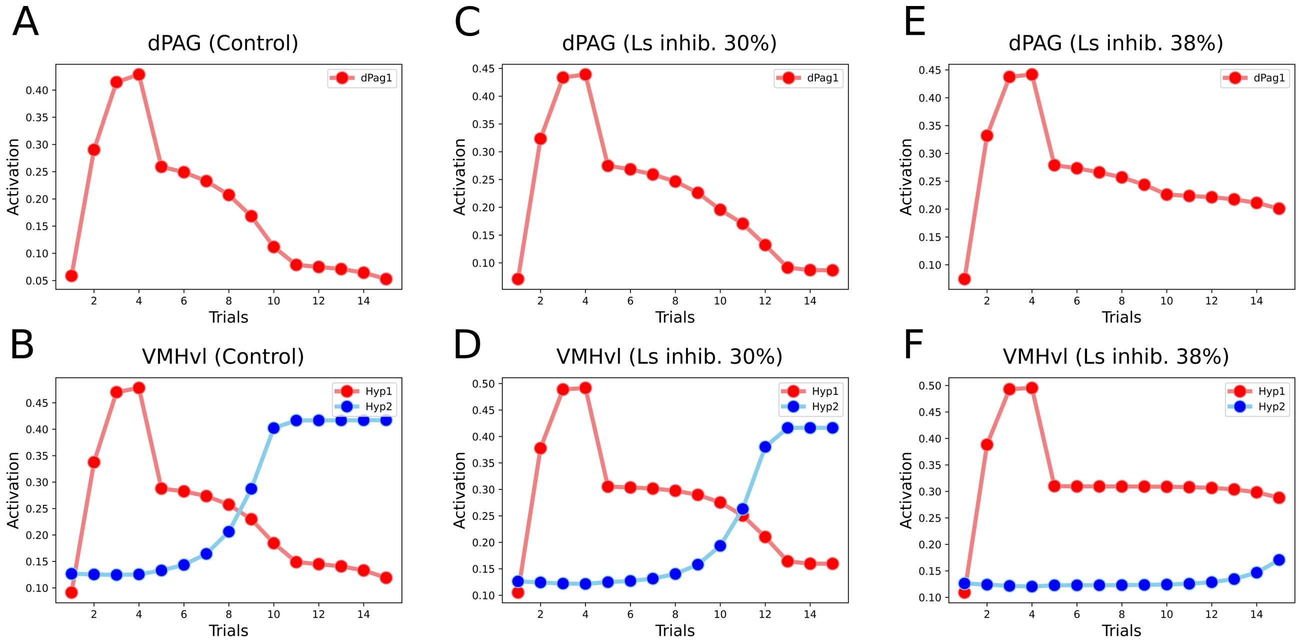

Figure S1. Effects of LS inhibition on social fear extinction. (A) dPAG and (B) VMHvl activity in control condition, without LS inhibition. (C) dPAG and (D) VMHvl activity in control condition, with a reduction of 30% of the LS output to VMHvl. (E) dPAG and (F) VMHvl activity in control condition, with a reduction of 38% of the LS output to VMHvl.

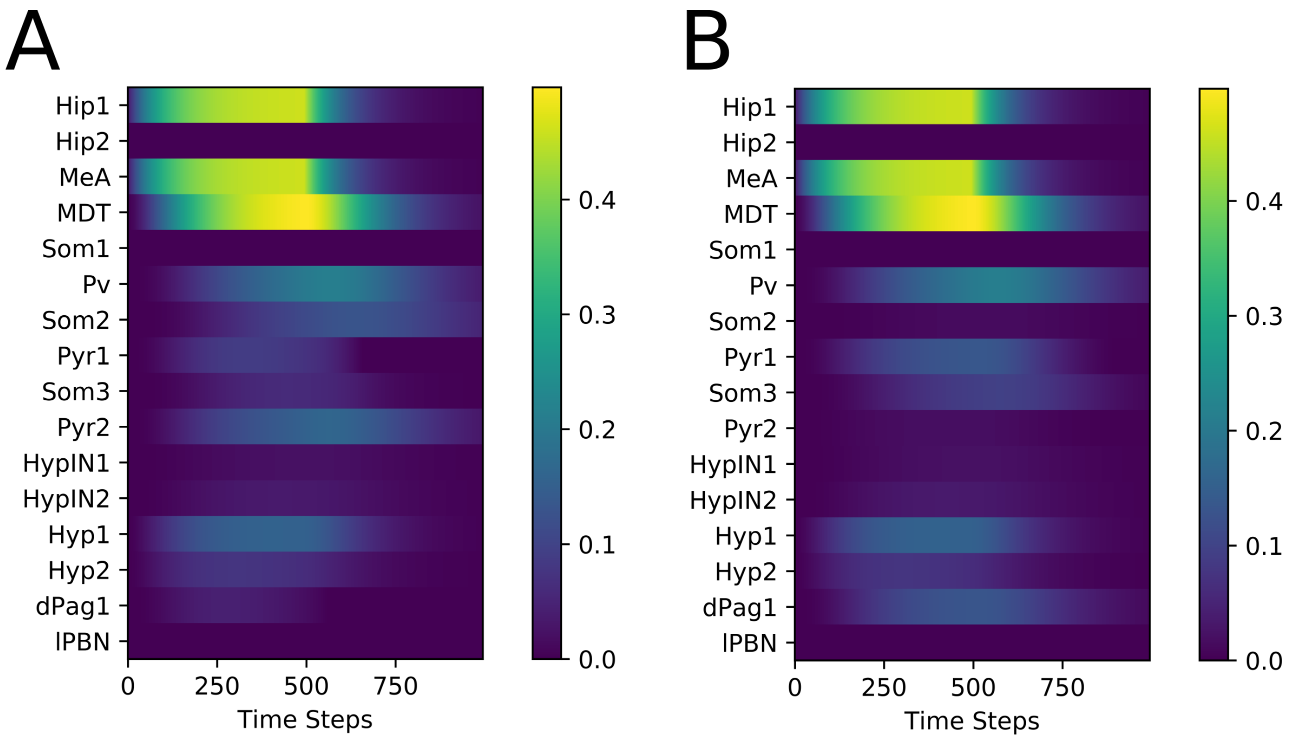

Figure S2. Inhibition of mPFC neurons projecting to the dPAG. Heatmaps showing the activity of the system during a single trial of exposure to a conspecific (activity in Hip1 and MeA) in two different conditions: the control (A) or when the Pyr2 unit is inhibited (B).

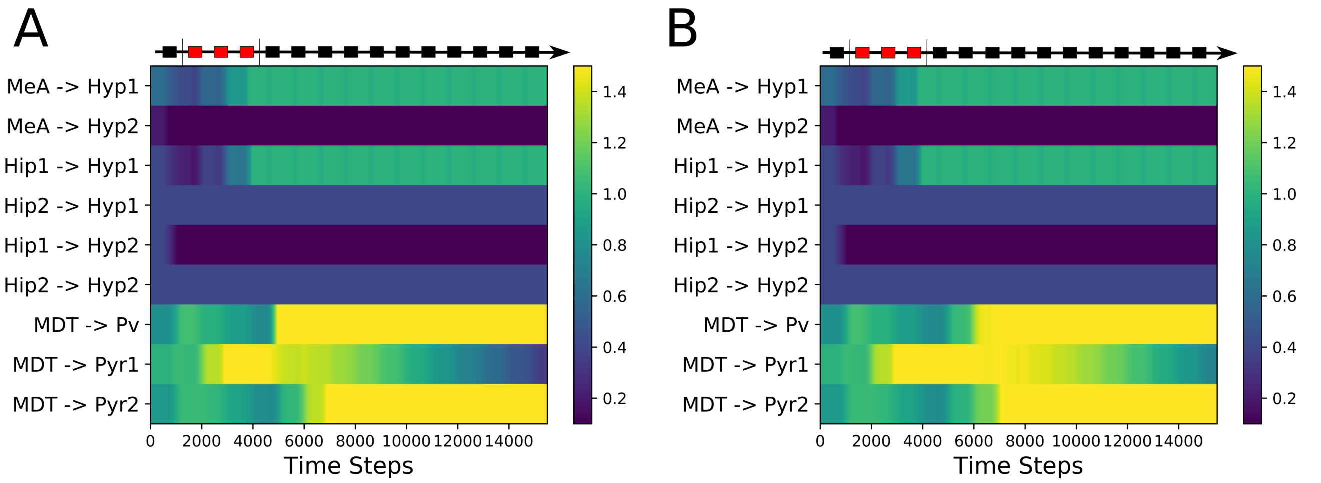

Figure S3. Effect of inhibitory units manipulation in mPFC on fear extinction. Heatmaps showing the weights changes of the system subjected to Pv unit stimulation (A) or Som1, Som2 and Som3 units inhibition (B).

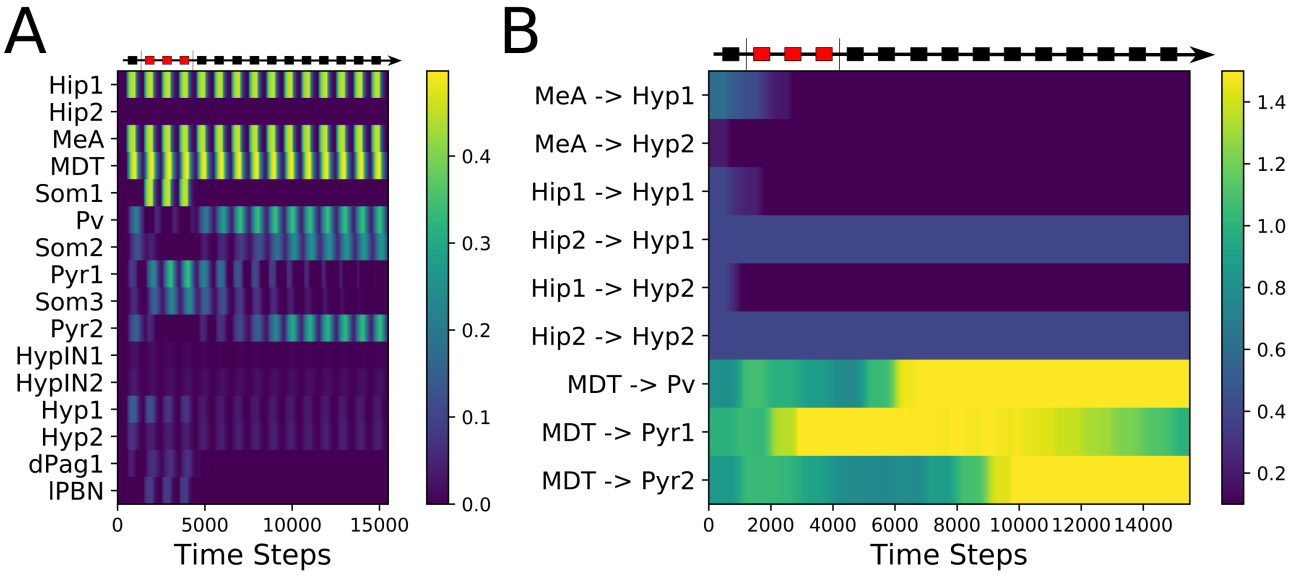

Figure S4. Inhibition of lPBN unit during conditioning. Heatmaps showing the activity (A) and the weights (B) of the system subjected to lPBN inhibition during conditioning.

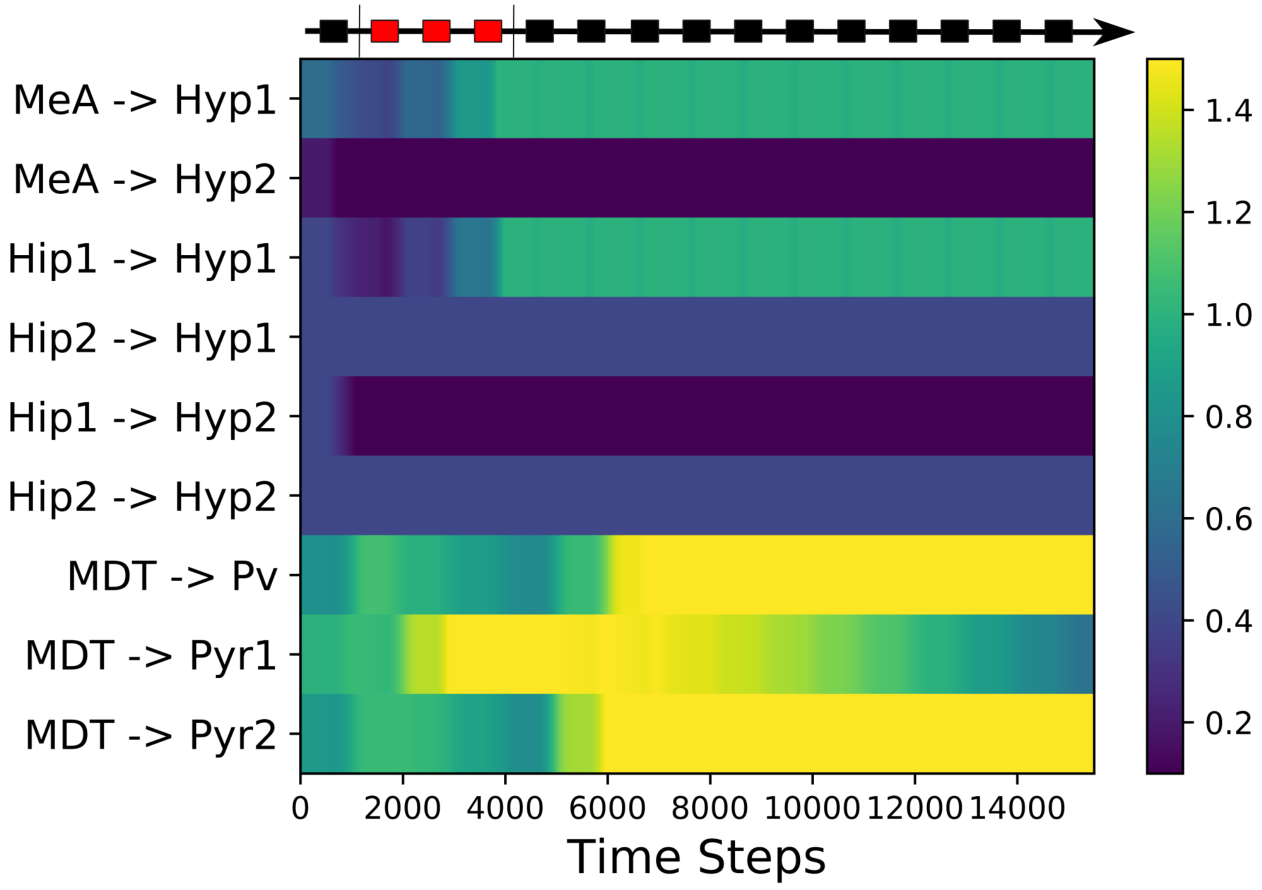

Figure S5. Stimulation of mPFC neurons projecting to the dPAG. Heatmaps showing the weights changes of the system subjected to stimulation the Pyr2 unit during the first trial of extinction.

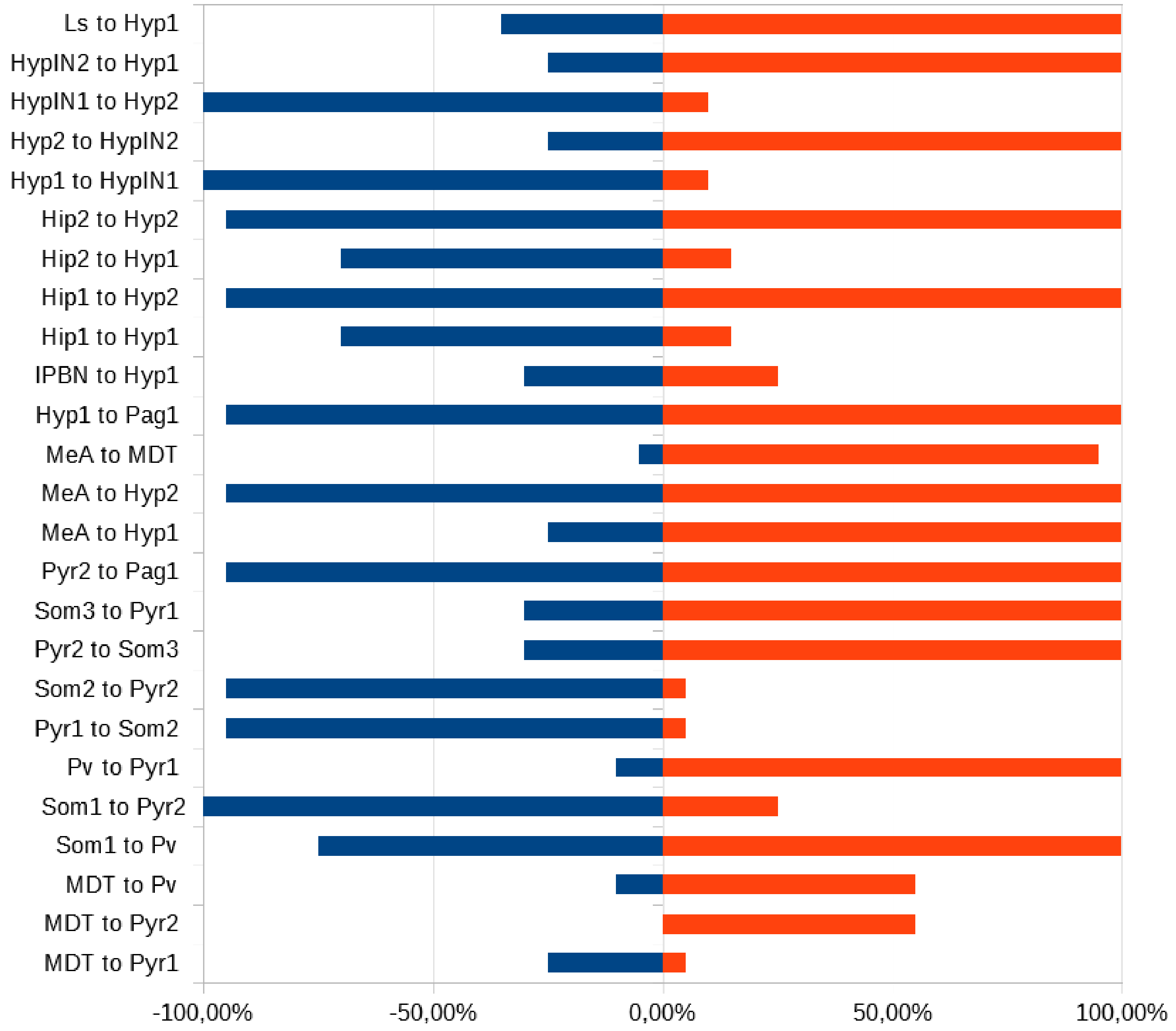

Figure S6. Results of the sensitivity analysis. For each connection, it is shown how much its weight can be decreased or increased before losing at least the fitting of one of the experiments

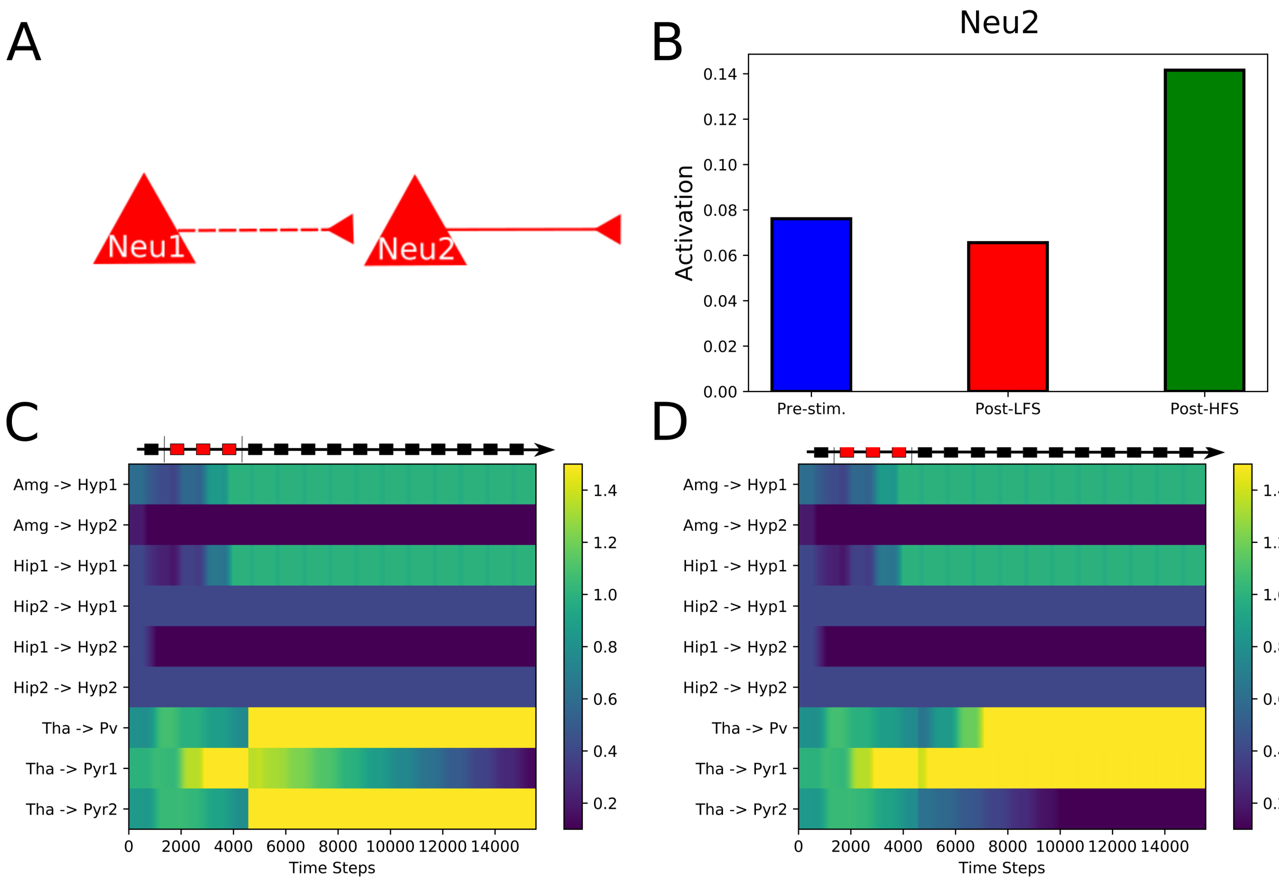

Figure S7. Effects of HFS and LFS of the MDT unit. (A) Scheme of two excitatory test neurons stimulated with HFS or LFS. The connection between the neurons is plastic. (B) Response of the postsynaptic unit Neu2 to a test stimulation delivered to the Neu1 unit before and after the HFS or LFS protocols. (C-D) Heatmaps showing the weights remodelling in the system during the delivery of the HFS (C) or LFS (D).

REFERENCES

Canteras, N., Simerly, R., and Swanson, L. (1995). Organization of projections from the medial nucleus of the amygdala: a phal study in the rat. *Journal of Comparative Neurology* 360, 213–245

Chang, C.-H. and Gean, P.-W. (2019). The ventral hippocampus controls stress-provoked impulsive aggression through the ventromedial hypothalamus in post-weaning social isolation mice. *Cell reports* 28, 1195–1205

Chiang, M. C., Nguyen, E. K., Canto-Bustos, M., Papale, A. E., Oswald, A.-M. M., and Ross, S. E. (2020). Divergent neural pathways emanating from the lateral parabrachial nucleus mediate distinct components of the pain response. *Neuron*

Collins, D. P., Anastasiades, P. G., Marlin, J. J., and Carter, A. G. (2018). Reciprocal circuits linking the prefrontal cortex with dorsal and ventral thalamic nuclei. *Neuron* 98, 366–379

Delevich, K., Tucciarone, J., Huang, Z. J., and Li, B. (2015). The mediodorsal thalamus drives feedforward inhibition in the anterior cingulate cortex via parvalbumin interneurons. *Journal of Neuroscience* 35, 5743–5753

Franklin, T. B., Silva, B. A., Perova, Z., Marrone, L., Masferrer, M. E., Zhan, Y., et al. (2017). Prefrontal cortical control of a brainstem social behavior circuit. *Nature neuroscience* 20, 260

Fulwiler, C. E. and Saper, C. B. (1984). Subnuclear organization of the efferent connections of the parabrachial nucleus in the rat. *Brain Research Reviews* 7, 229–259

Herry, C. and Garcia, R. (2002). Prefrontal cortex long-term potentiation, but not long-term depression, is associated with the maintenance of extinction of learned fear in mice. *Journal of Neuroscience* 22, 577–583

Krettek, J. and Price, J. (1977). Projections from the amygdaloid complex to the cerebral cortex and thalamus in the rat and cat. *Journal of Comparative Neurology* 172, 687–722

Krzywkowski, P., Penna, B., and Gross, C. T. (2020). Dynamic encoding of social threat and spatial context in the hypothalamus. *Elife* 9, e57148

Mitchell, A. S. and Chakraborty, S. (2013). What does the mediodorsal thalamus do? *Frontiers in systems neuroscience* 7, 37

Naka, A. and Adesnik, H. (2016). Inhibitory circuits in cortical layer 5. *Frontiers in neural circuits* 10, 35

Nelson, A. C., Kapoor, V., Vaughn, E., Gnanasegaram, J. A., Rubinstein, N. D., Murthy, V. N., et al. (2019). Molecular and circuit architecture of social hierarchy. *bioRxiv* , 838664

Nordman, J. C., Ma, X., Gu, Q., Potegal, M., Li, H., Kravitz, A. V., et al. (2020). Potentiation of divergent medial amygdala pathways drives experience-dependent aggression escalation. *Journal of Neuroscience* 40, 4858–4880

Risold, P. and Swanson, L. (1997). Connections of the rat lateral septal complex. *Brain research reviews* 24, 115–195

Saltelli, A., Ratto, M., Andres, T., Campolongo, F., Cariboni, J., Gatelli, D., et al. (2008). *Global sensitivity analysis: the primer* (New York, NY: John Wiley & Sons)

Silberberg, G. and Markram, H. (2007). Disynaptic inhibition between neocortical pyramidal cells mediated by martinotti cells. *Neuron* 53, 735–746

Silva, B. A., Mattucci, C., Krzywkowski, P., Murana, E., Illarionova, A., Grinevich, V., et al. (2013). Independent hypothalamic circuits for social and predator fear. *Nature neuroscience* 16, 1731–1733

Wong, L. C., Wang, L., D'amour, J. A., Yumita, T., Chen, G., Yamaguchi, T., et al. (2016). Effective modulation of male aggression through lateral septum to medial hypothalamus projection. *Current biology* 26, 593–604

Xu, H., Liu, L., Tian, Y., Wang, J., Li, J., Zheng, J., et al. (2019). A disinhibitory microcircuit mediates conditioned social fear in the prefrontal cortex. *Neuron* 102, 668–682

Zhou, T., Zhu, H., Fan, Z., Wang, F., Chen, Y., Liang, H., et al. (2017). History of winning remodels thalamo-pfc circuit to reinforce social dominance. *Science* 357, 162–168
